# Seasonal total methane depletion in limestone caves

**DOI:** 10.1038/s41598-017-07769-6

**Published:** 2017-08-16

**Authors:** Chris L. Waring, Stuart I. Hankin, David W. T. Griffith, Michael A. Kertesz, Victoria Kobylski, Neil L. Wilson, Nicholas V. Coleman, Graham Kettlewell, Robert Zlot, Michael Bosse, Graham Bell

**Affiliations:** 1 ANSTO Environmental Research, New Illawarra Rd., Lucas Heights, NSW 2234 Australia; 2 University of Wollongong, Centre for Atmospheric Chemistry, Wollongong, NSW 2522 Australia; 3 University of Sydney, Sydney Institute of Agriculture, Sydney, 2006 Australia; 4 University of Sydney, School of Life and Environmental Sciences, Sydney, 2006 Australia; 5 formerly CSIRO, Technology Court, Pullenvale, QLD 4069 Australia

## Abstract

Methane concentration in caves is commonly much lower than the external atmosphere, yet the cave CH_4_ depletion causal mechanism is contested and dynamic links to external diurnal and seasonal temperature cycles unknown. Here, we report a continuous 3-year record of cave methane and other trace gases in Jenolan Caves, Australia which shows a seasonal cycle of extreme CH_4_ depletion, from ambient ~1,775 ppb to near zero during summer and to ~800 ppb in winter. Methanotrophic bacteria, some newly-discovered, rapidly consume methane on cave surfaces and in external karst soils with lifetimes in the cave of a few hours. Extreme bacterial selection due to the absence of alternate carbon sources for growth in the cave environment has resulted in an extremely high proportion 2–12% of methanotrophs in the total bacteria present. Unexpected seasonal bias in our cave CH_4_ depletion record is explained by a three-step process involving methanotrophy in aerobic karst soil above the cave, summer transport of soil-gas into the cave through epikarst, followed by further cave CH_4_ depletion. Disentangling cause and effect of cave gas variations by tracing sources and sinks has identified seasonal speleothem growth bias, with implied palaeo-climate record bias.

## Introduction

Cave methane depletion is near universal for 44 caves reported^1–6^. A methanotrophic bacterial oxidation mechanism was first proposed to account for rapid methane loss over a few hours^1,2,5^ in opposition to a recently proposed radiolytic CH_4_ destruction mechanism^4^.

A steady-state model of *in-situ* depletion of methane by cave dwelling methanotrophs or radiolytic destruction by ions derived from radon decay might be expected from the year round constant cave air temperature ± 2 °C. However, an *in-situ* steady-state model is inconsistent with our observations of strong diel and seasonal bias in CO_2_, CH_4_ and other trace gases.

Cave CO_2_ variations are strongly anti-correlated with those of CH_4_. CO_2_ is enhanced relative to the external ambient atmosphere, to as high as 10,000 ppm in summer but remains near external ambient in winter. CO_2_ has several potential sources, including breath from cave visitors, ground air^7^, speleothem growth^8,9^ and karst soil gas^10^. CO_2_ sources that would be expected to show antithetical behaviour relative to CH_4_ are ground air^7^ and the overlying soil gas, where CO_2_ is increased by plant root and microbial respiration and CH_4_ is depleted by microbial oxidation.

## Dynamic cave ventilation

**Figure 1:**
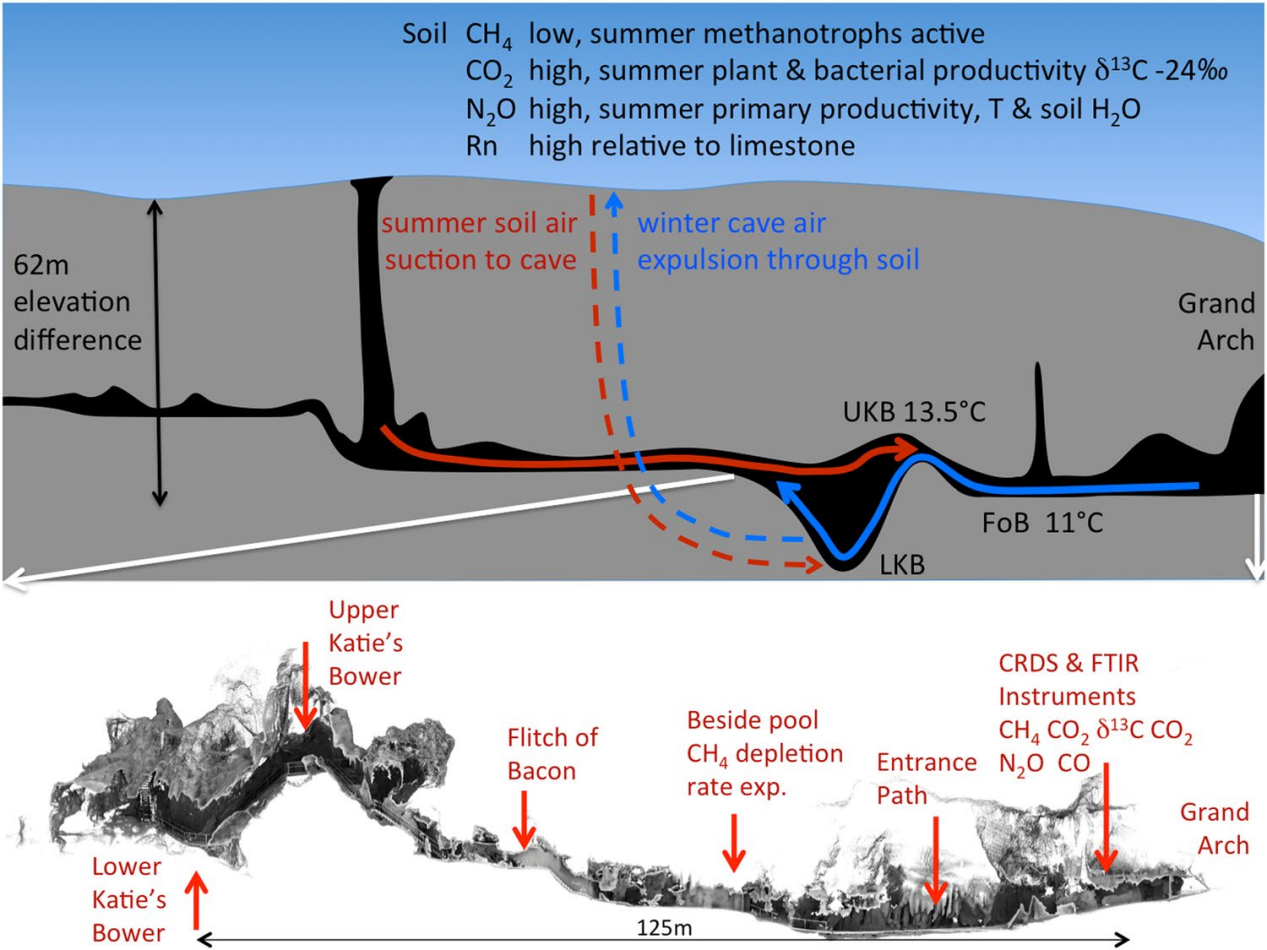
**Top panel** is a schematic cross-section through Chifley Cave showing the dominant convective air-flow directions for summer (red) and winter (blue). Air-flow along major passages may reverse direction daily in response to a reversal of the cave to external temperature difference, without a time lag (<15 mins). The slower passage of air from soil to cave along small cracks and fissures in summer has a longer time lag of 1–6 hrs between cave - external temperature reversal and the soil air peak in Lower Katies Bower (low CH_4_, high CO_2_, high N_2_O, high Rn). **Lower panel** is a scale diagram of continuous flow air sampling locations relative to the CRDS & FTIR instruments.

Convective cave ventilation^11^ driven by temperature contrast between the cave and the external environment also plays an important role in determining cave trace gas concentrations. Jenolan Caves, located approximately 100 km west of Sydney, Australia are representative of many mid-latitude cave locations exhibiting diurnal, synoptic and seasonal temperature cycles of the ambient atmosphere. These cycles create a contrast between the external temperature and the relatively constant cave temperature (11 °C ± 2 °C) to drive cyclical bi-directional convective ventilation through upper and lower cave openings (Fig. 1, Supplementary Fig. 1).

**Figure 2:**
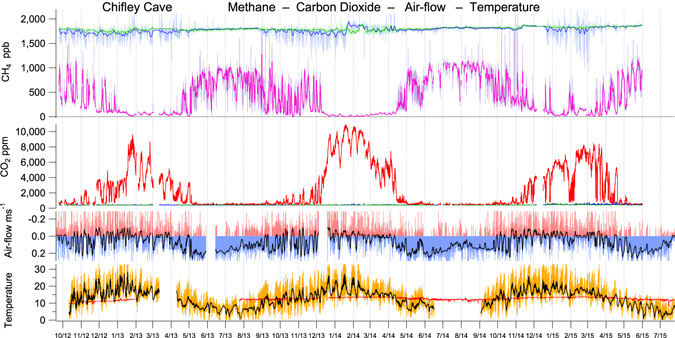
CH_4_ and CO_2_ CRDS continuous measurements (8 minute averages, recorded hourly) from LKB in Chifley Cave over 3 years from September 2012 to August 2015. Methane measurement error ± 20 ppb and carbon dioxide ± 5 ppm. Winter air-flow (blue infill) shows external ambient air drawn into Chifley Cave from the “Grand Arch” cave entrance has a greater velocity and longer duration than Summer air-flow (red infill) in the opposite direction. External weather has a direct immediate impact on cave CO_2_ & CH_4_ (eg. week 1 February 2015) linking cave trace gas composition to external weather and climate.

Our continuous data for CH_4_, CO_2_, δ^13^C-in-CO_2_, Rn, CO, and N_2_O trace gases from Chifley Cave (Fig. 1), represents a well-ventilated tourist cave hosting several tours per day. We also measured cave air-temperature, air-flow, air-pressure, drip-rate, and drip pH continuously for 3 years from September 2012 to July 2015. High precision gas analysers for CO_2_, CH_4_, CO, N_2_O and δ^13^C-in-CO_2_ (CRDS and FTIR, see methods) were installed approximately 20 m from the lower (Grand Arch) entrance and sequentially analysed air sampled once per hour drawn along tubing from four locations in the cave (Fig. 1) and 2 external reference locations. The karst soil overlying Chifley Cave was continuously monitored for soil temperature, soil water fraction and CO_2_, supplemented by hourly CH_4_ and δ^13^C-in-CO_2_ measured during a 10 day experiment to define soil function as a response to weather and gas composition as a potential source for cave air (Supplementary Fig. 2a,b).

The differential air density at different air temperatures inside the cave compared to external temperature drives bi-directional convective air-flow through the major passages, measured at the Flitch of Bacon (Fig. 1). The open passages provide the path for the bulk of cave air-flow. Air flows more slowly through minor passages & narrow fissures and ultimately through a multitude of cracks and through the soil. Summer air-flow is dominantly from the ridge overlying Chifley Cave including soil gas seepage with winter air-flow in the opposite direction (Fig. 2). On any one day throughout the year air-flow may change direction abruptly in response to changing external weather (temperature). In spring and autumn diurnal external temperature may dip below cave temperature and also rise above cave temperature (Fig. 2) causing a rapid (~15 min) change in air-flow direction along open passages measured at Flitch of Bacon (Fig. 1). The time lag between air-flow direction change and emerging CO_2_ peak in Lower Katies Bower is 1–6 hrs, giving an approximate soil to cave gas transit time. Soil to cave minimum distance is ~50–150 m compared to 268 m through major passages. The spring and autumn pattern of diurnal change in air flow direction and consequently large changes in CO_2_ Lower Katies Bower (1,000–5,000 ppm) is masked in summer and winter due to dominant flow directions. Normal soil gas flow into Chifley Cave is by wholesale mass transfer rather than gas diffusion. Rarely does external temperature equate to internal temperature for an extended period which may induce diffusion.

## Cave and karst soil CH_4_, CO_2_ and Rn links to air-flow & external weather

**Figure 3:**
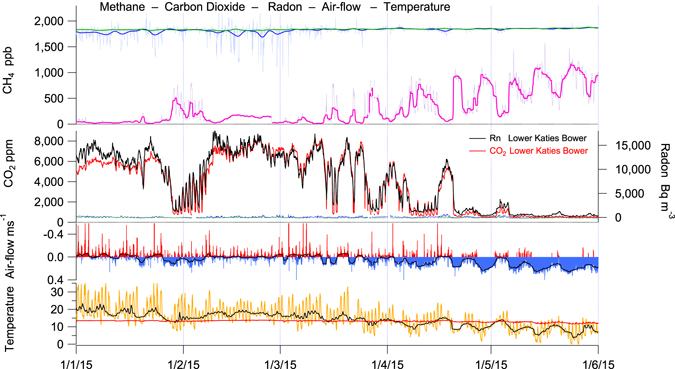
Chifley Cave, Lower Katies Bower trace gases CH_4_, CO_2_, and Rn, relative to cave air flow, cave and external temperature. The summer to winter period January 2015 – June 2015 illustrates the direct link between external temperature and cave CH_4_, CO_2_, and Rn through diurnal, synoptic & seasonal cycles.

**Figure 4:**
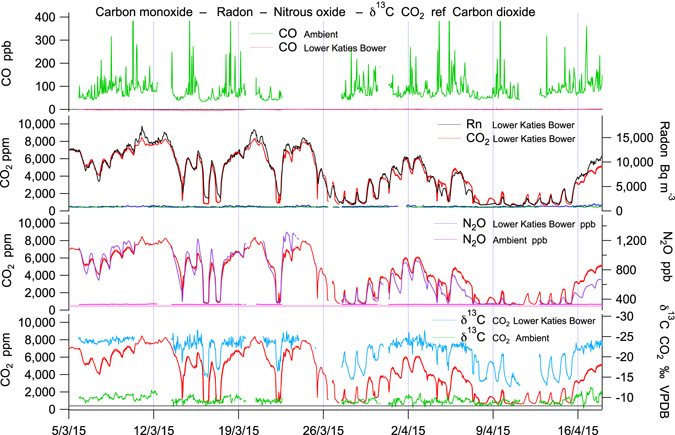
Late summer time series of CO, CO_2_, Rn, N_2_O, and δ^13^C – CO_2_ from Lower Katies Bower corresponding with correlation plots (5/3/15–18/4/15). CO is totally depleted (measurement error ± 5 ppb) within Chifley Cave relative to a variable background ambient concentration of 75–110 ppb. CO depletion is not seasonally variable. Rn, N_2_O and δ^13^C – CO_2_ (−24.8‰ VPDB) gas tracers each correlate strongly with CO_2_ and identify karst soil overlying Chifley Cave as the only likely source of high CO_2_ in the cave in summer (Figs 1 and 2). Late summer bi-directional air-flow shows large synchronous fluctuations in CO_2_ and gas tracers.

**Figure 5:**
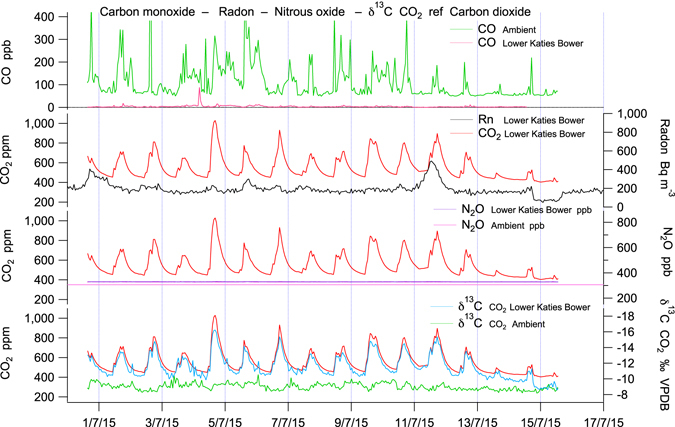
Winter time series of CO, CO_2_, Rn, N_2_O, and δ^13^C – CO_2_ from Lower Katies Bower corresponding with correlation plots (30/6/15–17/7/15). CO_2_ & Rn is 10x lower in winter. Rn and N_2_O soil gas tracers do not correlate with CO_2_. Air-flow is dominantly from the Grand Arch (Figs 1 and 2) diluting cave air with ambient air. Winter uni-directional air-flow with low CO_2_ induces speleothem growth (δ^13^C – CO_2_ = −19.5‰ VPDB, Fig. 6) and shows large synchronous fluctuations in CO_2_ not Rn & N_2_O soil gas tracers indicating a small cave source of CO_2_ from speleothem growth.

Methane in Lower Katies Bower (LKB) chamber within Chifley Cave was totally depleted from the normal atmospheric mole fraction of ~1,775 ppb to 11 ± 20 ppb in summer (Fig. 2, Table 1), correlating inversely with carbon dioxide, which sometimes exceeded 10,000 ppm in LKB in summer. In winter methane depletion in LKB was approximately half compared to external air, dropping to 550-1,150 ppb, while CO_2_ approached ambient air, ~570 ppm in LKB. Both CO_2_ and CH_4_ also displayed daily antithetic cycles of approximately one third of the full concentration range evident during autumn, winter, and spring. Diurnal CO_2_ and CH_4_ cycles were largely absent during summer. A synoptic cycle is clearly seen linking the passing of external weather systems to cave CO_2_ and CH_4_ (Figs 2 and 3). In addition to CH_4_, CO was also totally depleted in the cave relative to ambient air (Figs 4 and 5). Cave CO was always near zero and did not display seasonality.

**Table 1:** Summary of trace gases in Chifley Cave, measured in Lower Katies Bower during two typical weeks in summer and winter.

Chifley Cave	Lower Katies Bower		week	5/3/15–12/3/15	Winter	Winter	week	30/6/15–7/7/15
	Summer	Summer	Summer	Summer change			Winter	Winter change
	MIN	MAX	AVERAGE	cave–ambient	MIN	MAX	AVERAGE	cave–ambient
CH_4_ ±20 ppb	−2	21	11	>99% depletion	556	1,147	981	55% depletion
CO_2_ ±5 ppm	4,014	8,463	6,566	>14 × enrichment	446	1,029	568	1.3 × enrichment
δ^13^C–CO_2_ ±1‰ PDB	−24.9	−23.2	−24.2	−14.1‰ difference	−16.2	−9.9	−11.8	−2.7‰ difference
N_2_O ±5 ppb	659	1,196	989	3 × enrichment	328	331	329	no difference
CO ±5 ppb	−3	0	−2	>99% depletion	0	86	5	>90% depletion
Rn ±25 Bq m^−3^	3,264	13,824	9,999	>500 × enrichment	133	408	203	>10 × enrichment
Cave temperature	13.0	13.6	13.5	4.4 °C cooler	11.0	12.5	12.1	8.5 °C warmer
External temperature	7.4	29.1	17.8		−3.7	14.3	3.6	
Air-flow m s^−1^	−0.919	0.140	−0.027		−0.127	0.302	0.095	

A week is approximately the duration of a single synoptic cycle where the external temperature range and pressure remain stable causing a pattern of cave ventilation air-flow typical of the season. The change in the cave environment is relative to the synchronous trace gas measurement of the external ambient atmosphere using 8-minute averages taken hourly and averaged over approximately a week. Weekly averaging removes the diurnal cycle (MIN & MAX) from the reported change between cave and ambient atmospheres. There is a marked seasonal difference in the magnitude of depletion or enrichment for each trace gas reflecting the dominant air-flow direction with different sources of trace gases (Summer – soil & external ambient air, Winter – external ambient air). Comparison of weekly average cave temperature to weekly average external temperature differs in sign and magnitude seasonally. The difference in temperature between cave and ambient causes the seasonal reversal of dominant air-flow with consequent winter seasonal bias in average air-flow velocity and air-mass ventilated through the cave. Doubling the temperature difference has caused a trebling of average air-flow velocities.

Radon may emanate directly from limestone and cave sediments or be transported from overlying karst soil to the cave environment. Low uranium concentration in limestone and a short effective Rn diffusion path length favour a cave sediment (clay) or karst soil Rn source by a factor of ~100 over a limestone source^12^. This Rn source ratio was tested in Chifley Cave by experiments in which chambers were placed over cave surfaces and the chamber monitored for *in-situ* accumulation or depletion of Rn and CH_4_ (Supplementary Fig. 3). Synchronous measurement of Rn in sealed chambers covering a 0.23 m^2^ area of karst soil and 0.22 m^2^ of cave floor showed a karst soil to Chifley Cave Rn source ratio of 133 (Supplementary Table 1). Thus, Rn is a selective tracer for external karst soil air entering Chifley Cave, rather than being produced *in-situ* then diluted by winter ventilation^4,13^ with external air. Chifley Cave (Fig. 3), Ojo Guarena Cave^4^, Spain (ref.^4^, Supplementary Fig. 2) and Hollow Ridge Cave, Florida (ref.^13^, Fig. 4) also show highly correlated, relatively high, yet variable Rn and CO_2_ in summer and low stable Rn and CO_2_ in winter.

Rn geochemically discriminates between soil air and ground air^7^. Residual karst soils are composed of minerals that have not dissolved during limestone weathering, typically clays and Fe-oxides with organic carbon added by surface plants and soil bacteria. If ground air is distinct from soil air it is not expected to be in contact with the clays and Fe-oxides in the soil to inherit characteristically high Rn from a soil source.

Aerobic karst soil above Chifley Cave was depleted in methane (500–800 ppb) (Supplementary Fig. 2b) relative to atmospheric methane 1,750–1,800 ppb, typical of many aerobic karst and forest soils^6,14,15^. During a 10 day summer monitoring period, soil CH_4_ was observed to decrease rapidly, within 24hrs, in response to a small 4 °C soil temperature increase approximating the inverse soil diurnal CO_2_ response to temperature (Supplementary Fig. 2b).

**Figure 6:**
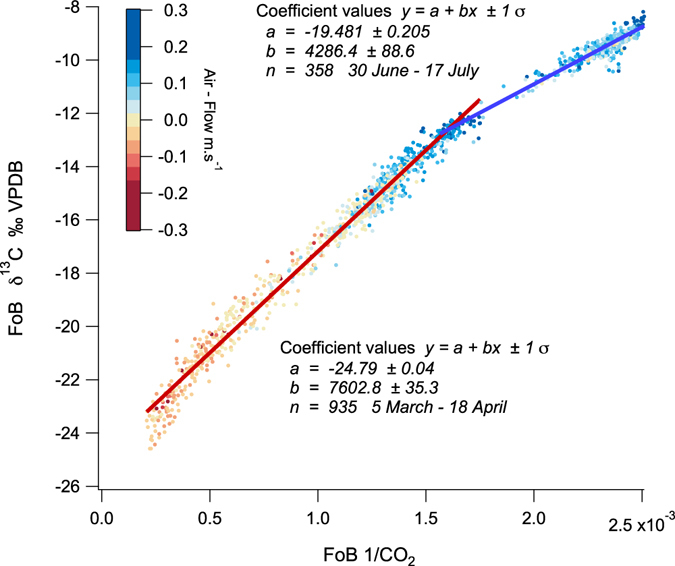
A Keeling plot of δ^13^C – CO_2_ vs 1/CO_2_ from Flitch of Bacon. for two time periods; autumn 5/3/15–18/4/15, least squares line fit (red), n = 935 and winter 30/6/15–17/7/15, least squares line fit (blue), n = 358. Data points are colour coded to represent air-flow direction and velocity. The Keeling plot intercept provides the δ^13^C – CO_2_ of the CO_2_ source component mixing with air. The source of high CO_2_ in LKB during autumnal (April) bi-directional airflow has δ^13^C – CO_2_ = −24.8‰ VPDB. The source of cave CO_2_ during winter with near uni-directional airflow has δ^13^C – CO_2_ = −19.5‰ VPDB, representing speleothem growth CO_2_ degassing within the Cave (δ^13^C drip water ^−^HCO_3_ (aq) −10.0‰ + fractionation^16^^−^HCO_3_ (aq) - CO_2_ (g) at 11 °C −9.48‰ = −19.5‰ VPDB).

Karst soil above Chifley Cave showed a variable seasonal CO_2_ pattern reflecting temperature and moisture-dependent soil primary productivity, sourced from plant root respiration and microbial organic matter degradation. Karst soil CO_2_ measured *in-situ* had a stable isotope label δ^13^C of −24‰ VPDB (Supplementary Fig. 2b) and did not vary significantly over the summer measurement period in response to large external temperature or soil moisture variations. Convective wholesale mass transfer of CO_2_ from soil to cave does not isotopically fractionate CO_2_. Potential fractionation by slow diffusion through porous media along a chemical gradient is not consistent with the measured air-flow regime and is not supported by matching soil and cave δ^13^C – CO_2_ measurements. In Chifley Cave with a summer ventilation pattern drawing air from above the cave with a soil source, the same (δ^13^C −24‰ VPDB) isotopic signature was observed in Lower Katies Bower (−24.8‰ Keeling intercept Fig. 6). This isotopic CO_2_ signature provides a second stable soil air tracer (after Rn) distinct from the well-mixed external atmosphere at Jenolan (CO_2_ δ^13^C −9.1 to −10.1‰ VPDB). A winter air-flow pattern in the opposite direction drew cool ambient air with low CO_2_, ~425 ppm into Chifley Cave from the Grand Arch cave entrance. Low CO_2_ in the cave atmosphere induces outgassing^16^ of CO_2_ from drip-waters during speleothem growth (Fig. 6, CO_2_ δ^13^C −19.5‰ VPDB) raising CO_2_ to ~560 ppm.

**Figure 7:**
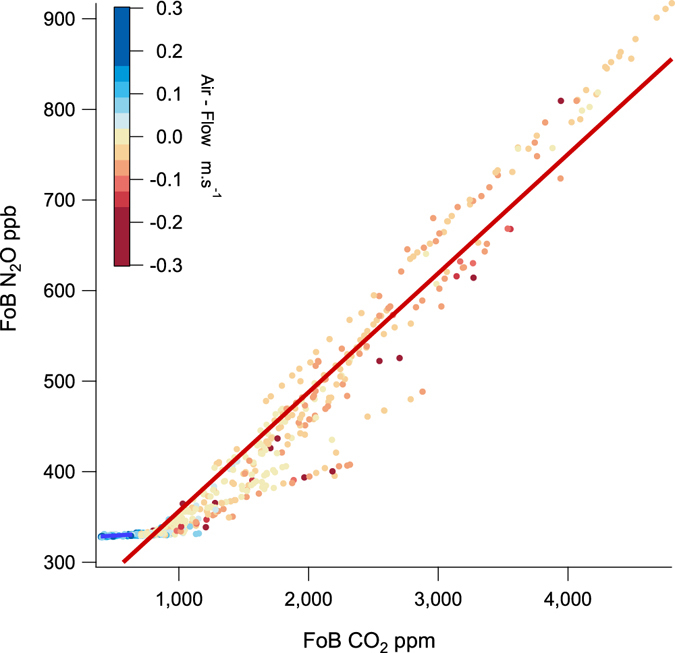
Correlation plot of N_2_O vs CO_2_ from Flitch of Bacon (0.5 × 2.0 m passage) for two time periods; autumn 5/3/15–18/4/15, least squares line fit (red), n = 935 and winter 30/6/15–17/7/15, least squares line fit (blue), n = 358. Data points are colour coded to represent air-flow direction and velocity. Only air from above Chifley Cave (ambient + soil air, red) contains N_2_O greater than ambient, indicative of soil air. Air-flow in the opposite direction (blue) is tightly clustered around ambient composition with no addition or depletion of N_2_O within Chifley Cave.

N_2_O provided a third selective tracer for soil air in the cave. N_2_O is produced in soils by nitrifying and de-nitrifying bacteria^17,18^ and is not produced or consumed inside caves from limestone or cave sediments (Fig. 7). N_2_O thus acts as a conservative tracer of soil gas. N_2_O is also closely correlated with CO_2_ and Rn (Figs 4 and 5) with an N_2_O:CO_2_ ratio of around 0.1 ppb ppm^−1^, typical of other soils^19^. The unchanging N_2_O:CO_2_ ratio of around 0.1 ppb ppm^−1^ between literature values for soils and cave measurements suggests no other source of N_2_O or CO_2_, such as ground air^7^, on the path between soil and cave is necessary to explain Chifley Cave data. Figure 7 shows the close summer N_2_O - CO_2_ correlation sourced from soil with air-flow magnitude & direction shown in shades of red. When N_2_O is measured inside Chifley Cave in winter (Fig. 7, blue) typically with air-flows in the opposite direction and an ambient atmospheric composition there is no soil gas contribution. N_2_O is fixed at atmospheric composition (Table 1, N_2_O = 329 ppb range ± 2 ppb error ± 5 ppb).

Collectively, Rn, N_2_O and high concentration ( > 5,000 ppm) CO_2_ with an isotopic label of δ^13^C −24‰ VPDB unequivocally identified karst soil as the source of these trace gases in air, emerging in LKB by convective suction of air in summer. However, a karst soil source of air with methane levels of 400–800 ppb does not fully explain total CH_4_ depletion in LKB in summer. Further *in-situ* cave CH_4_ depletion is inferred.

## Methanotrophic bacteria

Methanotrophic bacteria living on limestone and sediment cave surfaces, or in the overlying karst soil, may cause the observed depletion of methane in cave air. Methane oxidation by bacteria in soils and sediments is usually carried out by *Gammaproteobacteria* and *Alphaproteobacteria*^20^, though there is also recent evidence for methanotrophy by members of the *Verrucomicrobia*^21^. In natural soil environments that are not exposed to elevated levels of methane, the upland soil “USC-cluster” methanotrophs dominate. These grow well at atmospheric-methane concentrations but respond slowly to changes in methane availability, and have proved difficult to cultivate in the laboratory^22^. External karst soils overlying Gaden cave^6^, New South Wales showed that the methanotroph population in the upper 10 cm was dominated by *Alphaproteobacteria*, while *Gammaproteobacteria* were prevalent at 30 cm depth, and methanotrophic *Verrucomicrobia* were present at both depths. However, internal cave formations & sediments bacteria were not reported^6^.

This is the first study to investigate the presence, spatial and temporal distribution of cave methanotrophs. The presence of the bacterial methane monooxygenase gene *pmoA* gene was measured in internal cave soils and on cave wall and floor surfaces within Chifley Cave. The *pmoA* gene was detected in all soils and sediments tested, but not in samples swabbed from wet speleothem surfaces, indicating the presence of methanotrophic bacteria in the sediments, but not commonly on the cave walls. Similar results were obtained from other caves in the Jenolan complex, and from similar karst caves at Wombeyan, 100 km south of Jenolan. Sequencing of the *pmoA* gene diversity from four sites within Chifley Cave revealed a total of 88 new methanotroph OTUs (operational taxonomic units), using 86% amino acid identity as a cut-off for OTU delineation. These sequences fell into four known *pmoA* clades and two previously undetected clades (Supplementary Fig. 4, Supplementary Table 2). Sequences falling within the four known clades were related to sequences from methanotrophs that had previously been found in samples from upland soils, and which are predicted to comprise organisms adapted to methane metabolism at atmospheric concentrations^23^. There was no evidence for verrucomicrobial methanotrophs in Chifley cave, but it should be noted that the *pmoA* genes of this group are very poorly characterized to date.

The total bacterial population in the soils was 10^7^−10^9^ 16S rRNA gene copies per gram soil, and did not change significantly during the year. Bacteria were highest in the dampest sediments (Pool and Flitch of Bacon), with tenfold lower populations in the deeper cave regions (Katie’s Bower). The proportion of methanotrophs in the total bacterial population was highest in the deeper cave regions, Lower Katies Bower. By contrast, the proportion of methanotrophs in these sediments (including those belonging to both *Gammaproteobacteria*, and *Alphaproteobacteria*, but largely excluding those of the so-called “upland soil cluster”) showed considerable variation in population during the year, making up around 4% of the total bacterial population in the summer months, and 0–2% in the winter months. (Supplementary Fig. 5). The population of “upland soil cluster” methanotrophs was relatively stable throughout the year, as previously reported for type II methanotrophs in other environments^20^.

Initial rates of methane depletion from laboratory microcosms containing Chifley Cave sediments were 0.8–3.9 nmol g^−1^ h^−1^, (Supplementary Table 3) confirming cave methanotrophic organisms actively oxidize methane. Complete depletion of 1,000 ppm methane was observed within 20 days, with no depletion seen for sterilized samples. Similar results were obtained at atmospheric methane concentrations (Supplementary Fig. 6a). Methane is depleted from laboratory air, 2 ppm, in the experiment reaction chamber at a rate of 0.51 mg CH_4_ m^−2^ h^−1^, five times faster than methane oxidation in aerobic upland soils^15^ which is rarely higher than 0.1 mg CH_4_ m^−2^ h^−1^. Comparative cave chamber experiments^24^ utilising a small 0.22 m^2^ sealed area of Chifley Cave rock & sediment surface had a CH_4_ depletion rate of 0.2 nmol m^−2^ s^−1^ (Supplementary Fig. 6b).

## Causal mechanism

Initial reporting of methane depletion in Jenolan Caves^1^ proposed bacterial consumption to explain the observed rapid loss of methane over a few hours. At St Michaels Cave, Gibraltar^2^, isotopic measurement of cave air depleted in CH_4_ shows^13^C enrichment in remaining CH_4_ to give δ^13^C up to −15‰ VPDB from an initial −47‰ in ambient air. This demonstrates an inverse correlation of CH_4_ abundance and isotopic^12^C depletion^2,6^ characteristic of Rayleigh distillation^25^ by biological kinetic fractionation^26,27^ consistent with methanotrophy.

An alternate radiolytic mechanism of radon generating atmospheric ions, causing methane oxidation has recently been proposed to explain a spatial correlation between low methane and relatively high ion concentrations in Spanish caves^4^. Methanotrophy was dismissed for these Spanish caves based on the absence of detected methanotrophs in remote subterranean locations and an interpreted inability to sustain methanotrophs’ metabolic requirements; this is contrary to ecophysiological features of microorganisms in natural ecosystems^28,29^.

To test the competing hypotheses for cave CH_4_ depletion we conducted laboratory experiments with and without methanotrophs and Rn (Supplementary Fig. 6a, Exp. 1 & 2). We presume Rn is the source of elevated ions observed in Spanish caves and measured an approximate proportional relationship; 1 Bq m^−3^ ≈ 10 ions cm^−3^. In these experiments no CH_4_ loss was observed at 1,000 times the Rn concentration in Spanish caves^4^ (ref.^26^ and Supplementary Fig. 6a, Exp. 1). Further increasing the radiolytic dose equivalent to an extreme 17 kGy, 2.7 × 10^6^ times cave Rn^4^, reduces CH_4_ in air by 19%. The same small reaction chamber, populated with 250 g of Chifley Cave mud, sediment & methanotrophs (methane monooxygenase *pmoA* gene 1.5 × 10^6^ copies g^−1^ sediment) consumed methane from 2,040 ppb in laboratory air to 293 ppb over 5.75 days (Supplementary Fig. 6a Exp. 2a). A control experiment under the same conditions in which the reaction chamber was sterilised to kill methanotrophs showed no methane depletion after 6 days (Supplementary Fig. 6a Exp. 2b). These experiments together demonstrate both a direct link to methanotroph depletion of CH_4_ and no link to Rn-initiated depletion.

Chemical kinetic arguments further confirm that the maximum possible reaction rates for destruction of CH_4_ by atmospheric ions, sourced from Rn at the observed concentrations are several orders of magnitude too slow to account for the observed destruction lifetime of CH_4_ of a few hours (see Supplementary information).

We conclude that methanotrophs are solely responsible for cave methane depletion and ions derived from Rn play no significant part in the cave CH_4_ sink.

## Cave methane depletion process

From our data for Chifley Cave we resolve that a three-step process is responsible for seasonal total CH_4_ depletion.

The seasonal ventilation difference between summer suction of karst soil gas into Lower Katies Bower compared to winter expulsion of cave air from LKB provides a mechanism for seasonally different sources of air in LKB (Fig. 1). External air temperature has a rapid, compounding effect on cave CO_2_ and CH_4_ by influencing primary productivity of the source soil gas composition and modulating the strength of convective air-flow for transport from soil to LKB. Diurnal, synoptic and seasonal cyclic variations in observed trace gas concentrations are due to temperature-driven convective ventilation patterns.

**Figure 8:**
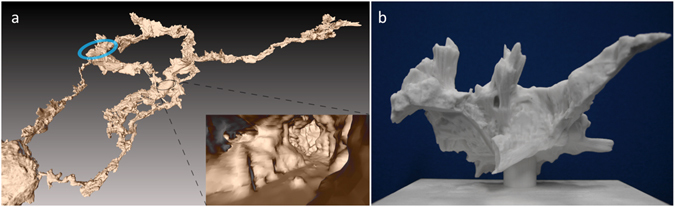
(**a**) Excerpt of high-resolution (3 cm) 3D mapping data^30,44^ for Jenolan Caves. This 2D image shows the details of passage connection from Grand Arch (left corner) through Chifley and Imperial Caves. Cut-away shows internal detail of 3D digital model, illustrating the complex volumetric and surface area relationships used to calculate air – cave surface interaction for bi-directional convective flow paths. Blue circle positions Lower Katies Bower. (**b**) Physical 3D printed model of Lower Katies Bower produced from high-resolution 3D mapping data^30,44^. Figure 1 schematically illustrates the thermal stratification in Lower Katies Bower under different convective air-flow regimes causing changes in air residence time.

Total methane depletion in summer is a consequence of initial partial depletion in the overlying karst soil by aerobic methanotrophs^14,15^ followed by slow interaction with cracks and fissure cave surfaces along the transport pathway to emerge in LKB. Further cave CH_4_ depletion occurs *in-situ* by methanotrophy on cave surfaces. Path length bias (Supplementary information text, Supplementary Fig. 7, Supplementary Table 4) and greater air residence time (Figs 1 and 8a,b) due to localised micro-temperature stratification in LKB may also contribute to the extreme summer methane depletion.

A simple measure of the temperature contrast between cave air and the external atmosphere coupled with the vertical difference in elevation between upper and lower cave openings gives an estimate of the strength of bi-directional convective cave ventilation (Table 1). Caves may also vary in convective ventilation response due to differing air path length and tortuosity^30^. Seasonal bias in convective cave ventilation strength and direction is likely to follow latitude and climatic zones^11^ with an increasing proportion of soil gas evident in low-mid latitude caves. Soil source gas composition, particularly CO_2_ and CH_4_ will vary with soil microbial primary productivity. Quantification of the processes described above give a guide to likely estimates of trace gases in caves.

The Chifley Cave methanotrophs reported here, which grow in cave soils at atmospheric methane concentrations (Type II methanotrophs), may be typical of many caves but are not unique to this cave or cave system. We have identified similar levels of methanotrophs in the Orient, Lucas, and Temple of Baal caves at Jenolan, and in the Wolondilly and Junction caves at Wombeyan Caves. The 88 new methanotrophic phylotypes identified in Chifley Cave are therefore likely to be representative of a larger group of limestone cave methanotrophs that have not yet been studied in detail, and are enriched in these environments. Extreme bacterial selection due to the absence of alternative carbon sources for growth in the cave environment has resulted in methanotrophs constituting 2–12% of the total bacteria present.

Caves and karst soils constitute an unrecognised^31^ aerobic soil methane sink^14,15^ and may contribute to uncertainties in aerobic soil sink climate feedback dynamics.

The direct links between dynamic methanotrophy and soil primary biological productivity dependent upon temperature and precipitation are observed and amplified in the cave system. When extrapolated to aerobic soils these insights may help to explain the observed anomalies in global atmospheric CH_4_ growth^32^. Aerobic soil response to climate change remains uncertain with CH_4_ feedback contributions from temperature and particularly precipitation strongly effecting aerobic soil CH_4_ sink dynamics. An example of this sensitivity is the reversal of seasonal aerobic karst soil CO_2_ and CH_4_ maxima and minima between Jenolan Caves and Wellington Caves^6^ 175 km to the North West, where primary biological productivity is limited in summer by soil water availability.

An exception to universal limestone cave methane depletion is noted^33^ for Movile Cave, Romania where Type I methanotrophs have also been described associated with a cave atmosphere at 1–2% methane^33,34^. Movile Cave is fed by hydrothermal groundwater rich in H_2_S, and this distinguishes it from other karst caves since the sulphide supports an unusual *in-situ* chemoautotrophic ecosystem. Microbial primary productivity acts as the food source for 48 species of cave-adapted terrestrial and aquatic invertebrates in Movile Cave^33^. By contrast, microbial life in most limestone caves is limited by the oligotrophic nature of the environment and organisms rely on reduced compounds in air, such as methane or carbon monoxide, or on chemoautrophy, oxidizing reduced metal ions present in the rock^35^.

Speleothem growth is favoured by low CO_2_ in the cave atmosphere. Definition of the soil source (Rn, N_2_O, δ^13^C CO_2_ tracers) and high concentration of CO_2_ with its isotopic label drawn into Chifley Cave in summer without change (soil & cave, δ^13^C −24‰ PDB) suggests speleothem growth inhibition. Winter speleothem growth is evident (Fig. 6) leading to the inference of strong seasonal bias in speleothem growth for convection-ventilated caves. Projection of this result in Chifley Cave to other caves and impact on palaeo-climate records is conditional upon the specific speleothem location in the cave ventilation pattern driven by local external weather and climate.

## Methods

### Cave gas measurement method

We designed our study to take advantage of technology advances in high precision field-deployable CRDS and FTIR spectrometers to continuously measure trace gases (CH_4_, CO, N_2_O, CO_2_ and δ^13^C-CO_2_) in a cave for the first time. This approach overcomes problems of 1. data paucity for spot sampling (ie monthly 12 samples per year) which cannot resolve diurnal and synoptic variations compared to hourly continuous 8,760 measurements per year per location × 6 2. the problem of individual samples taken sequentially during a sampling field trip not being an accurate snapshot in time due to changing cave ventilation (<1 hour) 3. Spot sampling of cave environments is also inherently difficult because of the human sampler breathing and potentially effecting results. Our trace gas data come from four cave locations, Lower Katies Bower (LKB) 120 m from the Grand Arch and Upper Katie’s Bower (UKB), Flitch of Bacon (FOB), Entrance path) (Fig. 1) together with two external reference locations in the ambient atmosphere outside the cave. A large cave chamber, Katies Bower, has two measurement points subject to different air-flow patterns, Lower Katies Bower at the base and Upper Katies Bower at the entrance – exit point in the main flow path. Flitch of Bacon is a 0.5 × 2.0 m narrow passage in the direct flow path.

### Cavity ring down spectrometer (CRDS)

CO_2_, CH_4_, H_2_O and δ^13^C-in-CO_2_ were measured with a CRDS spectrometer (Picarro Inc., Santa Clara, CA, USA, model G1101i with CH_4_ cell upgrade, CFFDS-35) installed with a programmable multi port valve (MPV) sequencer for switching between 16 possible inlet ports. Sample air was drawn continuously through all sample lines from the 4 locations in Chifley Cave and 2 external locations Ports 1–6 by a Vacuubrand ME2 vacuum pump at 1–5 L/min, then passed to an exhaust line and vented outside the cave. The CRDS instrument draws sample at 25 ml/min via the MPV, which is connected by 1/8” Tee-union to the side of all 6 sample lines and also 7th reference gas line. Non-return valves were fitted to all sample lines to prevent possible back-flow from the lowest resistance path or shortest sample line. The MPV was programmed to analyse gas from each sample line sequentially after a dwell time of 10 minutes for cave air and 5 mins for external air. A reference gas instrument air cylinder was also measured in the hourly sequence. CRDS instrument raw data recorded at 8 sec intervals was averaged to 1 minute intervals and the first 2 minutes after port switching was discarded for cell flushing. Further data averaging reduced the data to one representative, 8 minute average, sample per hour. A manufacturer installed CRDS instrument data correction procedure for spectral CO_2_ and CH_4_ interferences was augmented by an additional empirical H_2_O correction after extensive testing at low CH_4_ concentrations and high H_2_O (details available on request). After averaging and post-processing H_2_O correction, data were calibrated. 5 calibration gases were analysed at the National Measurement Institute, by gas chromatograph with methanizer-FID detector. The composition was determined by comparison with primary gas standards prepared gravimetrically using a Sartorius CC10000S mass comparator, with buoyancy correction based on the cylinder volume and air density at the time of each measurement. 3 of the 5 calibration gases measured by NMI were commercially available zero air cylinders certified to contain less than 100 nmol/mol trace gas other than N_2_ and O_2_.

These three zero air gases measured by NMI reported 23, 39, 32 nmol/mol CH_4_, compared to a sample of summer air from Chifley Cave, Lower Katies Bower which contained 10 nmol/mol. Measurement error for low CH_4_ calibration gases measured NMI by gas chromatography was 5 nmol/mol. Repeated measurement (8 minute average) of the instrument air gas cylinder in the hourly cycle had a 1σ precision of 15 ppb. Summation of calibration gas error and CRDS instrument precision gives a CH_4_ measurement error of 20 ppb.

### FTIR trace gas analyser

From March 2015 until the end of the experiment in July 2016 an FTIR trace gas analyser was deployed in parallel to the CRDS, sampling air from Upper and Lower Katies Bower, Flitch of Bacon and one external location through the same inlet lines in the same 1 hour cycle. The FTIR analyser was built at the University of Wollongong and is functionally similar to the Spectronus trace gas analyser (Ecotech Pty Ptd, Knoxfield, Vic Australia) and described in Griffith *et al*.^36^. The FTIR measured CO_2_ δ^13^C in CO_2_, CH_4_, CO and N_2_O in air with precision of the order of 0.1% for all species (1% for CO) and 1‰ for δ^13^C in CO_2_. The analyser was calibrated in the laboratory against four WMO-scale-traceable standards in the ambient air mole fraction ranges, and in the cave system against a single ambient level standard and against the high level standards described above for the CRDS.

The FTIR was also used to perform the in-cave chamber experiments. Air from the surface enclosures described in the main text was circulated in a closed loop between the surface enclosure and the FTIR, typically for 10–20 minutes alternating with flushing of the enclosure with ambient cave air for 10–20 minutes. Loss rates of CH_4_ in the enclosure were determined from the time-series of FTIR measurements. The flux calculations followed the methods given by Phillips *et al*.^24^.

### In-cave sensors

Environmental sensors were placed above the 2.0 × 0.5 m passage-way at Flitch of Bacon (FOB) drip water site near Chifley Cave exit to Grand Arch. Data was recorded at 15 minute intervals on a Datataker DT80 Series 2 data logger. Air-flow direction and velocity data were obtained using a Gill Instruments Windsonic 2D sonic anemometer Option A. Air temperature data was obtained using a Vaisala HMP50 sensor prior to 24-Jul-13 and a Vaisala HMP60 sensor installed after 24-Jul-13. Carbon dioxide concentration was collected using a Vaisala GMM220 transmitter and Vaisala GMP222 carbon dioxide 0–7000ppm probes until 4-Dec-2013, with a data gap occurring between 11-Oct-13 to 21-Feb-14 due to a GMP222 probe failure and replacement. Radon concentration data was collected at 1 hour intervals in the cave using a Saphymo Alphaguard P30 radon monitor configured in diffusion mode from 29-Aug-2014 to 17-Aug-2015 and located adjacent to the Picarro air intake for lower Katies Bower.

### Soil sensors

Soil sensors were installed on the surface of the hill above Chifley cave in the area surrounding the Plughole cave entrance. Soil sensor data was recorded at 15 minute sample intervals on Datataker DT82I Series 3 data logger. Soil CO2 concentration was obtained using a Vaisala GMM220 transmitter with a Vaisala GMP221 Carbon dioxide probe 0–3%. The GMP221 probe was installed in a 20 cm deep augered hole inside a Vaisala 211921GM in-soil adapter. Soil moisture and temperature data was obtained using a Stevens digital hydraprobe II installed in a separate augered hole 20 cm deep. Air temperature at the soil monitoring site was obtained using a Vaisala HMS82 intercap humidity and temperature transmitter in mini stevenson screen mounted at 2 m elevation on pole adjacent to soil probes. Radon concentration data was collected at 1 hour intervals using a Saphymo Alphaguard PQ2000 Pro radon monitor configured in flow mode. Air was pumped though the radon monitor at 1 L/min from a soil flux chamber located within 5 metres of the soil sensors.

### Weather station

Weather data was collected using a Monitor Sensors weather station installed in forest 50 north of the plughole cave entrance. Data was logged using a Monitor Sensors SL5 uSmart data logger at 30 minute intervals until to 12-Mar-13, and hourly thereafter. Air temperature was obtained using a Monitor sensors uSmart TA1 ambient temperature sensor. Rainfall data was collected using a Monitor sensors uSmart RG2 0.2mm tipping bucket rain gauge (TBRG) until 20-Mar-2014 and a Hydrological Services TB4 0.2mm TBRG with onboard ML1-FL data logger after 29-May-2014.

### Cave microbial diversity and quantification

Bacterial populations in cave sediments were classified by sequencing of the methane monooxygenase *pmoA* gene, using sediment DNA as template. Sediment samples (0–5 cm) were sampled aseptically, while swab samples were taken using a cotton swab dampened in sterile water then trimmed to fit into a 1.5 ml Eppendorf tube. Sediment and swab samples were stored at −20 °C until required, and total DNA was extracted using commercial kits (Mobio Powersoil), following the manufacturer’s instructions.

Methane monooxygenase diversity in the soil DNA was determined after amplification with the *pmoA* primers A189/A682^37^. Tag encoded-pyrosequencing analysis was done from the A189 primer, using Roche 454 FLX TitaniumTechnology (Research and Testing Laboratories, Lubbock, Texas). Raw sequence data was trimmed of low-quality bases using the Fungene pipeline^38^, and reads shorter than 150 base pairs were removed. Chimeric sequences were removed using USEARCH 6.0. Amino acid translations of sequences were obtained using FrameBot^39^, and sequences exhibiting less than 40% identity to a *pmoA* database (Fungene pipeline) were removed. Amino acid sequences were aligned using HMMER3 and phylogenetic trees were constructed in MEGA 5.2^40^.

Total bacteria and methanotrophic bacteria in cave sediments were quantified by real-time PCR, using sediment DNA as template and determining the abundance of the 16 S rRNA gene and the methane monooxygenase *pmoA* gene respectively. 16 S abundance was determined using the 341 F/806 R primer set^41^, and individual *pmoA* clades were quantified using specific gene probes: A189/mb661 (*pmoA*)^42^; A189/Gam634r (USC-γ clade)^23^; A189/CL1603R (Cluster 1 clade)^23^. The quantification standards used were either purified amplicons from cave DNA, or a plasmid containing the *pmoA* gene of *Methylococcus capsulatus* Bath^43^.

### Methanotrophic consumption of methane in cave sediments

Consumption of methane by sediments was measured by incubation of soil samples (5 g) in 120 ml serum bottles, corrected to a 10% (w/w) moisture content with deionized water. Methane was injected (1000 ppm), and the samples were incubated statically at room temperature. Methane disappearance was measured by gas chromatography on an HP-PLOT Q capillary column with helium as the carrier gas, using an oven temperature of 200 °C, and a flame ionisation detector. Dead controls were autoclaved prior to the initial methane injection.

### Laboratory radon exposure experiments

#### Experiment 1

To study radon (ion) – air (CH_4_) interaction a 10 L gas bag (SKC Flexfoil) was infused with radon from a sealed source (PYLON 2000A) providing an initial radon concentration of 1.25MBq m^−3^ decaying to 0.70MBq m^−3^ after 3.2 days (AlphaGuard PQ2000).

#### Experiment 2

For methanotroph – air interaction we constructed a simple closed circuit loop; 1.6 L fixed volume reaction chamber with ~250 g Chifley Cave, Jenolan Caves, NSW sediment, and 5 L gas bag with air recirculated at 100 mL min^−1^. *Experiment 2b* includes sterilisation (^60^Co gamma, dose 25kGy). Methane was measured with a CRDS spectrometer (Picarro) and methanotrophs by qPCR (Supplementary methods).

Methane was measured with a CRDS spectrometer by removing approximately 200 ml air from the circuit for each measurement (Picarro Inc., Santa Clara, CA, USA, model G1101i CH_4_ cell upgrade, CH_4_ is water corrected & calibrated with 1σ precision, 20 ppb). Laboratory air was continuously measured for background methane concentration during the experiments.

An empirical proportional relationship between radon concentration and ion concentration (AlphaLab Air Ion Counter) in air was observed for a sealed 30 L container which approximates 1 Bq m^−3^ ≈ 10 ions cm^−3^. However, we noted significant variability (factor of ~5) of ion counter values with position, time (1–10 mins) and proximity of people ± static charge on clothing in laboratory measurements.

### Three-dimensional cave mapping

Three-dimensional models of the caves^44^ were acquired using a novel handheld laser scanning system^30^. Path lengths between the entrances and various points in the caves were calculated by applying the A* path planning algorithm^45^ to a volumetric octree model derived from the laser data.

### Data Availability

Additional full data for all plots in “Seasonal total methane depletion in limestone caves” is available; Waring, Chris L; Hankin, Stuart I; Griffith, David W T; Kettlewell, Graham (2017): Continuous 3-year record of cave methane, trace gases and environmental data collected in Jenolan Caves, Australia. PANGAEA, 10.1594/PANGAEA.878077 now available at the PANGAEA data repository.

## Electronic supplementary material

Supplementary Information; 2 text sections, Kinetics of radiolytic CH4 destruction and Assessment of potential mechanisms for seasonal CH4 depletion pattern, 7 Figures, and 4 Tables
