# Author Correction: Seasonal total methane depletion in limestone caves

**DOI:** 10.1038/s41598-018-23872-8

**Published:** 2018-04-11

**Authors:** Chris L. Waring, Stuart I. Hankin, David W. T. Griffith, Michael A. Kertesz, Victoria Kobylski, Neil L. Wilson, Nicholas V. Coleman, Graham Kettlewell, Robert Zlot, Michael Bosse, Graham Bell

**Affiliations:** 1 ANSTO Environmental Research, New Illawarra Rd., Lucas Heights, NSW 2234 Australia; 2 University of Wollongong, Centre for Atmospheric Chemistry, Wollongong, NSW 2522 Australia; 3 University of Sydney, Sydney Institute of Agriculture, Sydney, 2006 Australia; 4 University of Sydney, School of Life and Environmental Sciences, Sydney, 2006 Australia; 5 formerly CSIRO, Technology Court, Pullenvale, QLD 4069 Australia

Correction to: *Scientific Reports* 10.1038/s41598-017-07769-6, published online 16 August 2017

In the original version of this Article, the following text was omitted from the Data Availability section:

“Additional full data for all plots in “Seasonal total methane depletion in limestone caves” is available; Waring, Chris L; Hankin, Stuart I; Griffith, David W T; Kettlewell, Graham (2017): Continuous 3-year record of cave methane, trace gases and environmental data collected in Jenolan Caves, Australia. PANGAEA, 10.1594/PANGAEA.878077 now available at the PANGAEA data repository.”

In addition, the Supplementary Information file was incorrectly titled ‘Data in “ Seasonal total methane depletion in limestone caves” ﻿is available;﻿Waring, Chris L; Hankin, Stuart I; Griffith, David W T; Kettlewell, Graham (2017): Continuous 3-year record of cave methane, trace gases and environmental data collected in Jenolan Caves, Australia. PANGAEA, 10.1594/PANGAEA.878077’.

These errors have now been corrected in the PDF and HTML versions of the Article.

