# Supplementary material for: Seasonal total methane depletion in limestone caves
Source: Sci Rep. 2017 Aug 16;7:8314. doi: 10.1038/s41598-017-07769-6 (PMC5559484; doi:10.1038/s41598-017-07769-6)
Supplement: Supplementary file 1 — Supplementary Information; 2 text sections, Kinetics of radiolytic CH4 destruction and Assessment of potential mechanisms for seasonal CH4 depletion pattern, 7 Figures, and 4 Tables [file 41598_2017_7769_MOESM1_ESM.pdf]

Seasonal total methane depletion in limestone caves

Chris L. Waring[1*], Stuart I. Hankin[1], David W.T. Griffith[2], Michael A. Kertesz[3], Victoria Kobylski[3], Neil L. Wilson[3], Nicholas V. Coleman[4], Graham Kettlewell[2], Robert Zlot[5], Michael Bosse[5], Graham Bell[5]

1. *ANSTO Environmental Research, New Illawarra Rd., Lucas Heights, NSW 2234, Australia*
2. *University of Wollongong, Centre for Atmospheric Chemistry, Wollongong, NSW 2522, Australia*
3. *University of Sydney, Sydney Institute of Agriculture, Sydney, 2006, Australia*
4. *University of Sydney, School of Life and Environmental Sciences, Sydney 2006, Australia*
5. *formerly CSIRO, Technology Court, Pullenvale, QLD 4069, Australia*

* corresponding author: clw@ansto.gov.au

Supplementary Information

Kinetics of radiolytic CH_4 destruction

Kinetic arguments suggest radiolytic methane destruction is orders of magnitude too slow to explain cave methane depletion. At the observed ion concentrations in cave atmospheres, ion reactions cannot account for the rapid CH_4 loss observed in laboratory experiments and in Chifley Cave. Assuming that ion concentrations in cave air are maintained approximately constant by continuous radon decay, methane loss should follow pseudo first order kinetics

$$\frac{d[CH_4]}{dt} = k[X][CH_4] = k_1[CH_4]$$

where k is the second-order rate constant for the reaction of CH_4 and ions, [X] represents the concentration of atmospheric ions, and k_1=k.[X] is a pseudo-first order rate constant since [X] is approximately constant. In this case methane decays exponentially with the rate constant k_1 s^{-1}, lifetime $\tau = 1/k_1$ s and half-life $\tau_{1/2} = \ln(2)/k_1$ s

$$[CH_4] = [CH_4]_0 e^{-k_1 t}$$

From laboratory measurements we find a proportionality between Rn and ion concentrations, 1 Bq m^{-3} ~ 10 ions cm^{-3}, from which typical total ions concentrations in Chifley cave are 10^2 - 10^5 cm^{-3}. Fernandez-Cortes et al. found $4x10^4$- $2x10^5$ ions cm^{-3} in Ojo Guarena cave (ref 4). Only a small fraction of these ions are reactive primary ions such as N^+, O^+, N_2^+ derived from N_2 and O_2; primary ions rapidly coagulate with water vapour and other trace gases to form complex ions which are much less reactive. For reactive primary ions the maximum possible second order rate constant[1] k is ~ $1x10^{-9}$ cm^{-3} s^{-1}. Estimating *at most* 1% of total ions existing as reactive ions, a *maximum* value for k_1 is ~ 10^{-9} * 10^3 = 10^{-6} s^{-1}, corresponding to a *minimum* methane lifetime to ion-induced decay of 10 days. Thus the ion mechanism is too slow by at least an order of magnitude to account for the observed rate of methane loss in the cave with a lifetime of a few hours.

The CH_4 loss mechanism via $OH^•$ radicals produced from radon decay suggested by Fernandez-Cortes et al (ref 4) is also not kinetically feasible. The rate constant for the CH_4 + $OH^•$ reaction is ~ 6 x 10^{-15} cm^{-3} s^{-1} at cave temperature[1], requiring an $OH^•$ concentration of ~ 10^{10} - 10^{11} cm^{-3} to explain the observed rate constant of 10^{-4} s^{-1} . This is 10^6 times higher than mean daytime $OH^•$ concentrations in the atmosphere in sunlight. With the production rate of total $HO_x^•$ = $OH^•$+$HO_2^•$ radicals from radon decay[2] of 4 x 10^5 Bq^{-1}, observed cave radon levels of ~ 1,000 Bq m^{-3} (10^{-3} Bq cm^{-3}), and a lifetime for $OH^•$ radicals[3] of ~1 s, the steady state total $HO_x^•$ concentration from radon decay (only some of which is $OH^•$) would be of the order of 4 x 10^2 cm $^{-3}$, 8 orders of magnitude too low to explain the observed rate of CH_4 loss.

Assessment of potential mechanisms for seasonal CH_4 depletion pattern

In the absence of a significant seasonal temperature difference in Chifley Cave (Figure 2, Supplementary Figure 1) causing changes to methanotroph activity we consider other possible mechanisms to account for the strong seasonal CH_4 observed.

a Seasonal ventilation, - macro-convection

We ask, is a simple steady state model of *in-situ* methanotroph CH_4 depletion diluted with seasonally varied external air velocity compatible with observations? The simple answer is no, because convective air-flow through Chifley Cave is seasonally bi-directional with different sources. In winter Chifley Cave is diluted with external air alone, CH_4 is ~800 ppb. In summer soil air is also drawn into Chifley Cave to totally deplete methane.

Summer peak CO_2 observed in Lower Katies Bower, (~8,000 ppm, $\delta^{13}C$ -24 ‰ PDB) matches soil derived microbial and root respiration labelled with high Rn (> 2,000 Bq m^{-3}) and high N_2O (> 1,000 ppb) indicating an *exogenous* karst soil origin for air in Lower Katies Bower. Winter air in LKB (CO_2 ~600 ppm) does not match the overlying winter soil CO_2 concentration, 3,000 - 4,500 ppm indicating seasonally different sources of air in LKB caused by a sharp change in the dominant convective air-flow direction (Figure 2). Any single day may have a temperature reversal between external and cave temperatures, causing air-flow reversal and different proportional mixing and residence time for gas source tracers. External soil CH_4 depletion to 600 – 800 ppb, or exposure to cave surfaces (winter cave air CH_4 ~800 ppb) alone is insufficient to account for CH_4 approaching zero for the summer months (Figure 2).

b Convective ventilation & measurement asymmetry causing seasonal path length bias

There is a bias in the cave path length and exposed surface area from the lower Grand Arch opening to LKB measurement point (116 m, 4,496 m^2) compared to the upper Elder Cave opening (268 m, 19,714 m^2) (Figure 1). Different path lengths and interactive cave surface area to the measurement point in LKB may partially explain seasonal CH_4 depletion pattern where the dominant seasonal convective air-flow is reversed. The magnitude of the path length bias (3:7) or cave surface area bias (2:8) produced by *in-situ* methanotrophy does not account for the extreme seasonal methane depletion pattern observed (> 99% summer, 55% winter) (Supplementary data Table 4).

c Cave micro-temperature environment

Air path length bias may influence very minor diurnal anomalies to cave air temperature (< 1.5 °C) due to less areal interaction with the rock mass in winter. Summer air-flow in the opposite direction with a greater path length and time for thermal equilibration with the rock mass does not vary cave air temperature (Supplementary Figure 1). Thermal stratification of air in Katies Bower causes summer air flow to pass over the top of the chamber. Winter air-flow in the opposite direction may retain a small low temperature anomaly relative to the rock mass causing sinking into Lower Katies Bower, effectively flushing with external air. The greater residence time for *in-situ* methanotrophy in summer may also contribute to the pattern of extreme methane depletion.

d Methanotroph activity changes

The population of Type I methanotrophs was significantly higher in the summer months, suggesting that these may play a minor role in the complete disappearance of cave methane seen in this season.

Supplementary information references

1. Burkhol JB*, et al.* Chemical Kinetics and Photochemical Data for Use in Atmospheric Studies, Evaluation No. 18. Preprint at http://jpldataeval.jpl.nasa.gov (2015).

2. Ding H, Hopke PK. HO x production due to radon decay in air. *Journal of Atmospheric Chemistry* **17**, 375-390 (1993).

3. Monks PS. Gas-phase radical chemistry in the troposphere. *Chemical Society Reviews* **34**, 376-395 (2005).

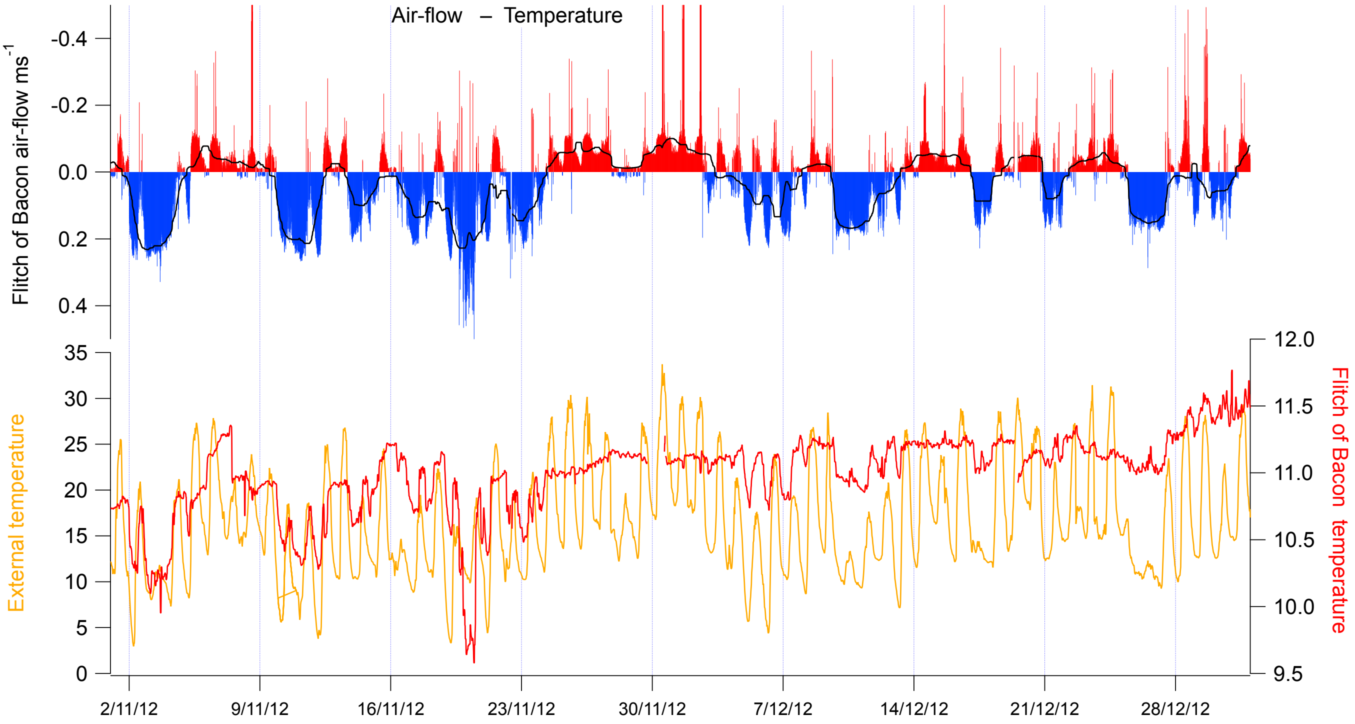

Supplementary Figure 1. An example of the direct link between external temperature (gold) and the consequent bi-directional convective air-flow in Chifley Cave.
Cave temperature at the Flitch of Bacon varies by less than 1.5 °C across seasons approaching thermal equilibration with the rock-mass. Daily winter FoB temperature excursions of 0.2 – 1.5 °C cooler than the monthly average due to ingress of cold external temperatures. The converse summer air-flow does not induce warm air excursions. A consequence of cool air anomalies relative to the rock-mass is regular winter flushing of low residence time air sinking into Lower Katies Bower (Figure 1). Summer air, thermally equilibrated with the rock-mass skims over the top of Katies Bower without mixing into Lower Katies Bower, increasing cave air residence time in Lower Katies Bower during summer with increased time for methanotrophs to consume methane. Temperature stratification reduces summer air mixing and increases residence time within large chambers such as Katies Bower.

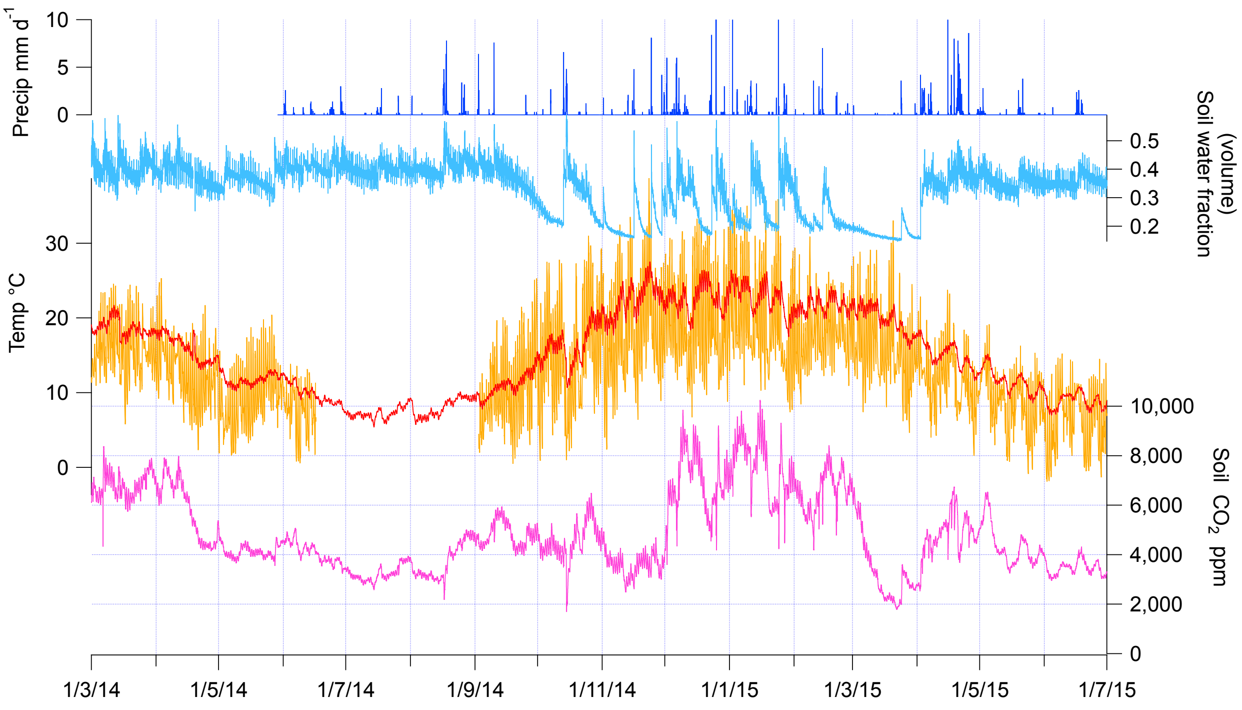

Supplementary Figure 2a. Soil function synchronous with last 15 months of cave monitoring data (Figure 2). External atmospheric temperature (gold) and soil temperature (red) shows strong diurnal and seasonal cycles. Precipitation (dark blue) short-term record over the preceding few weeks determines the soil water fraction. Soil CO_2 derived from plant root respiration and soil microbial activity is a proxy measure of primary biological productivity. Soil CO_2 concentration is sensitive to temperature and precipitation daily – weekly weather cycles.

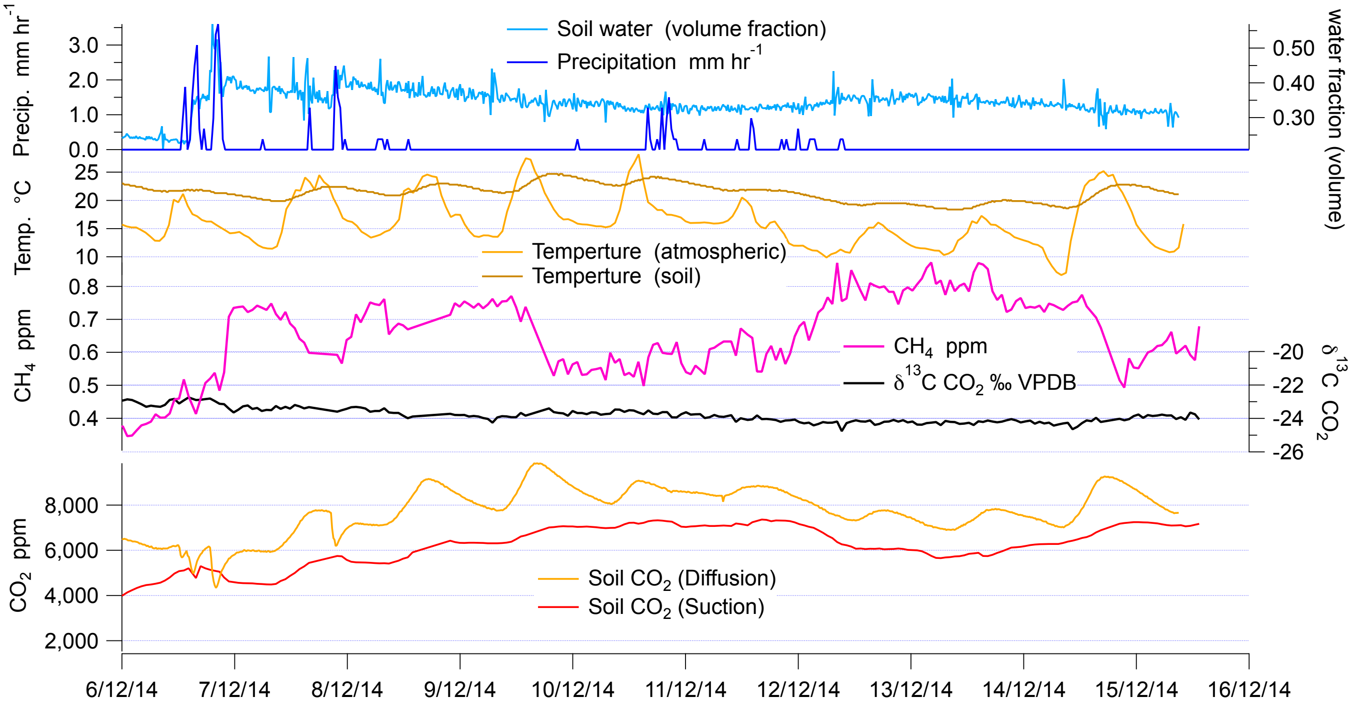

Supplementary Figure 2b. Detailed soil function for 10 days in summer, December 2014. Soil CO_2 is measured by diffusion (IR sensor) and by low volume gas suction to a CRDS instrument. Gas extraction for measurement is replaced by ambient air causing a small offset. Soil CH_4 shows an inverse relationship with CO_2 and primary biological productivity. Soil CH_4 is low (~600 ppb) when methanotroph activity is high under warm moist conditions. Soil CO_2 $\delta^{13}C$ -24 ‰ VPDB is invariant with respect to short-term weather conditions.

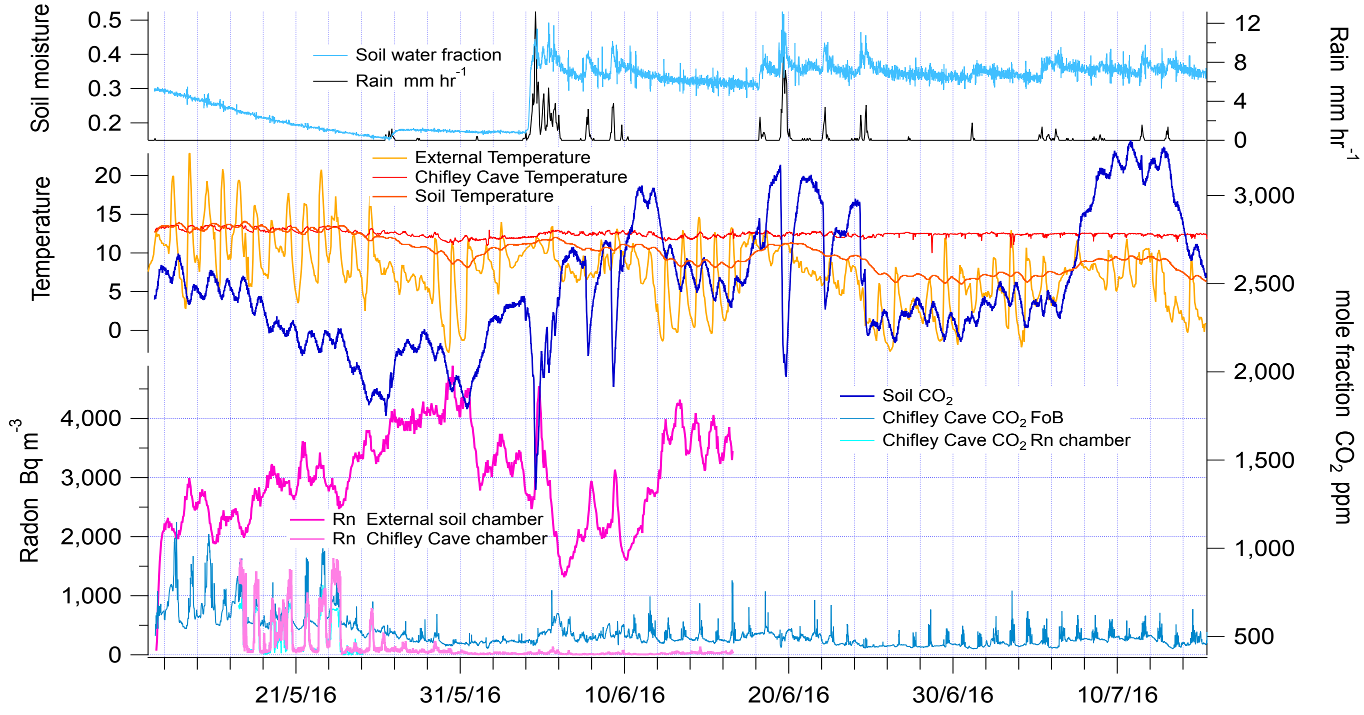

Supplementary Figure 3. Synchronous measurement of weather parameters (temperature & precipitation), soil (temperature, CO_2 & Rn) and cave (temperature, CO_2 & Rn) during the transition to winter. Soil CO_2 shows an initial decline in response to drying soil with individual small rain events causing an increase in dry soil CO_2. Large rain events restoring soil moisture cause an immediate large transient decline in CO_2. Soil Rn shows the inverse response, without temperature dependency. Cave CO_2 and Rn are decoupled from soil CO_2 and Rn when air-flow is from the Grand Arch in winter. In-situ accumulation of Rn in Soil chamber is > 100 times accumulation of Rn in similar size Chifley Cave chamber (Supplementary Table 1).

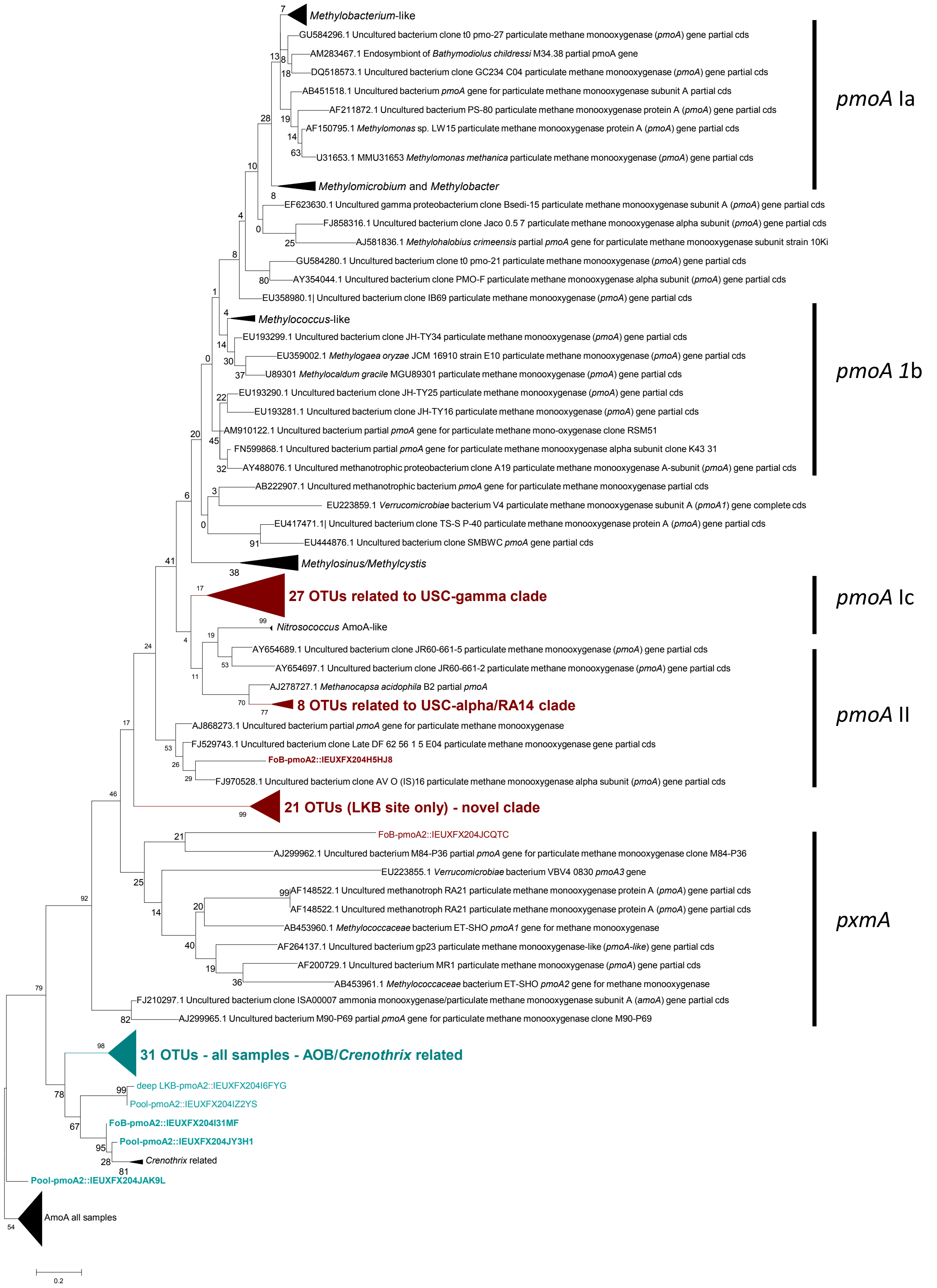

Supplementary Figure 4. Methanotrophic bacterial diversity in Chifley Cave soil samples. DNA samples from Upper and Lower Katie's Bower, Pool and FoB are shown. Sequences were obtained by tag encoded-454 pyrosequencing analysis. Operational taxonomic units are delineated at 93% amino acid identity of the encoded peptide. Reference sequences are from the Functional Gene Pipeline (fungene.cme.msu.edu). Red – Cave OTU's related to *pmoA*. Blue – Cave OTU's related to *AOB/Crenothrix* sequences.

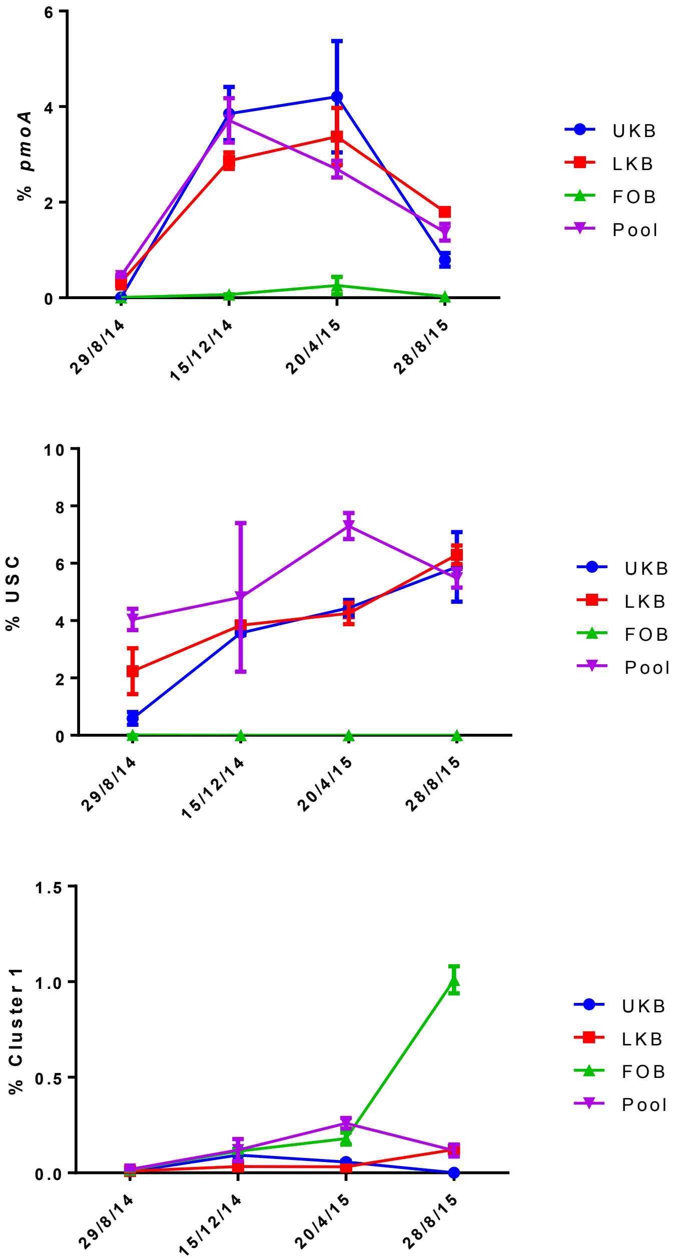

Supplementary Figure 5. Proportion of different groups of methanotrophs relative to the total bacterial population in cave sediments. Proportions were determined by quantitative PCR of the 16S rRNA gene (primers 341F/806R), the type I *pmoA* gene (primers A189/mb661), USC-gamma *pmoA* gene (primers A189/Gam634r), and Cluster 1 *pmoA* (primers A189/CL1-603r). Total bacterial population was 10^7 – 10^9 per gram soil. Samples were taken in triplicate, and error bars represent the standard error of the mean.

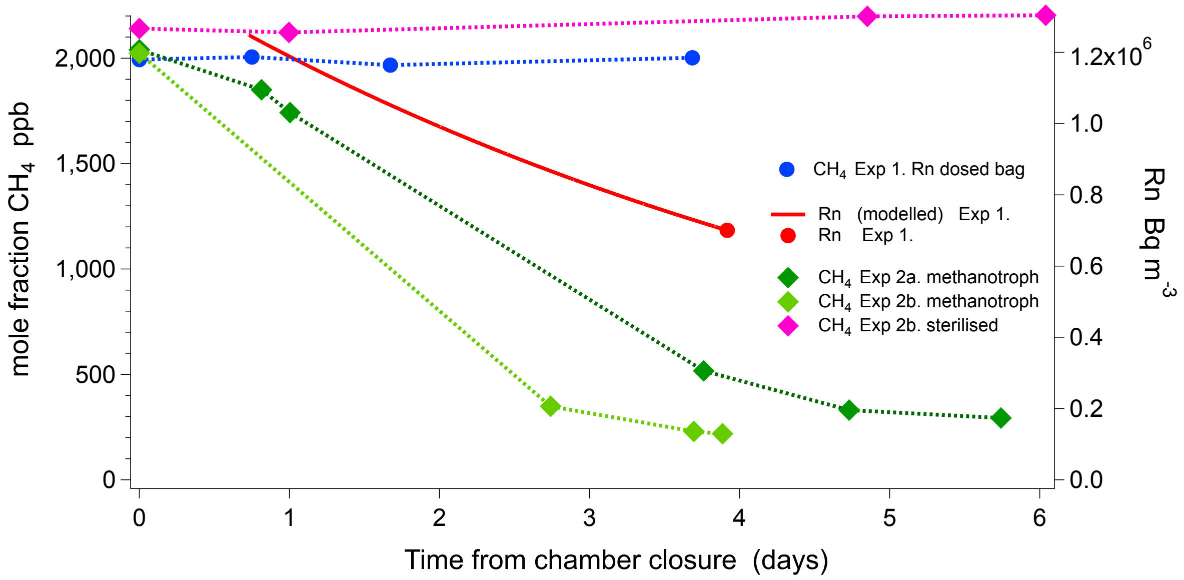

Supplementary Figure 6a. Methanotroph & Rn CH_4 depletion laboratory experiments
Experiment 1. Methane mole fraction in gas bag dosed with Rn (circle symbol), after 3.2 days exposure to radon at ~1,000 times the radon concentration of Ojo Guarena Cave[4] there was no significant change to methane mole fraction in the sample loop, initial 1,993 ppb, final 2,002 ppb.
Experiment 2. Methane mole fraction in closed circuit with methanotroph reaction chamber (diamond symbols). *Experiment 2a* After 5.75 days residence time in the 6.6L circuit containing ~250g sediment from Chifley Cave, methane concentration declined from an initial 2,040 ppb to a final 293 ppb. Analysis of sediment from Chifley Cave revealed high levels of the methane monooxygenase *pmoA* gene (1.5 x 10^6 copies per gram sediment). *Experiment 2b* Includes a second step sterilisation of methanotroph reaction chamber. After sterilisation, air in the reaction chamber maintained a constant methane mole fraction of 2,141 ppb – 2,203 ppb for 6.0 days.

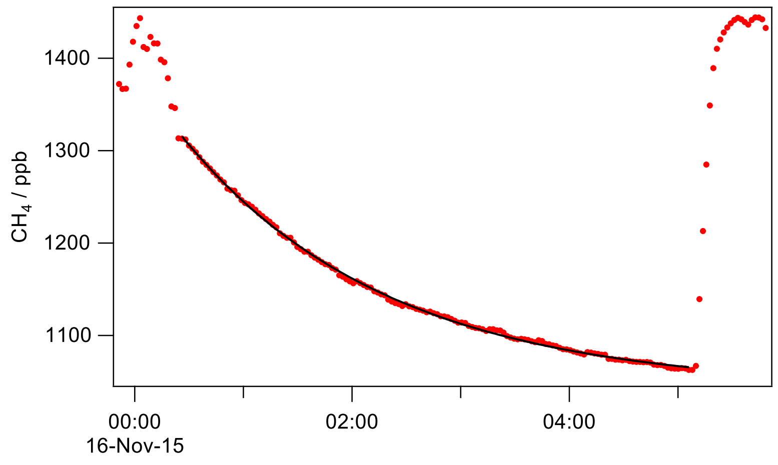

Supplementary Figure 6b. CH_4 *in-situ* depletion rate chamber experiment
A 0.22 m^2 area of cave rock & sediment surface within Chifley Cave was sealed with plastic sheeting to form a closed chamber with an effective height of ~0.1 m. Air from the chamber was re-circulated through the FTIR analyser in a closed loop. The chamber loop was periodically flushed with cave air then the closed loop sealed and air re-circulated for 1-3 hours with continuous measurement of CH_4 and CO_2. The CH_4 decay is fitted to a linear + exponential equation[35] which describes a linear surface uptake rate allowing for the fact that the chamber is not perfectly sealed and exchanges air with the external cave air. The leak exchange time is approx. 2 hours (causing the curvature away from linear CH_4 decrease) and the CH_4 uptake rate (initial slope) is typically 0.04±0.01 ppb s^{-1} or ~150 ppb hr^{-1}. For a 0.1 m effective chamber height, this corresponds to an uptake rate at the surface of 0.2 nmol m^{-2} s^{-1}.

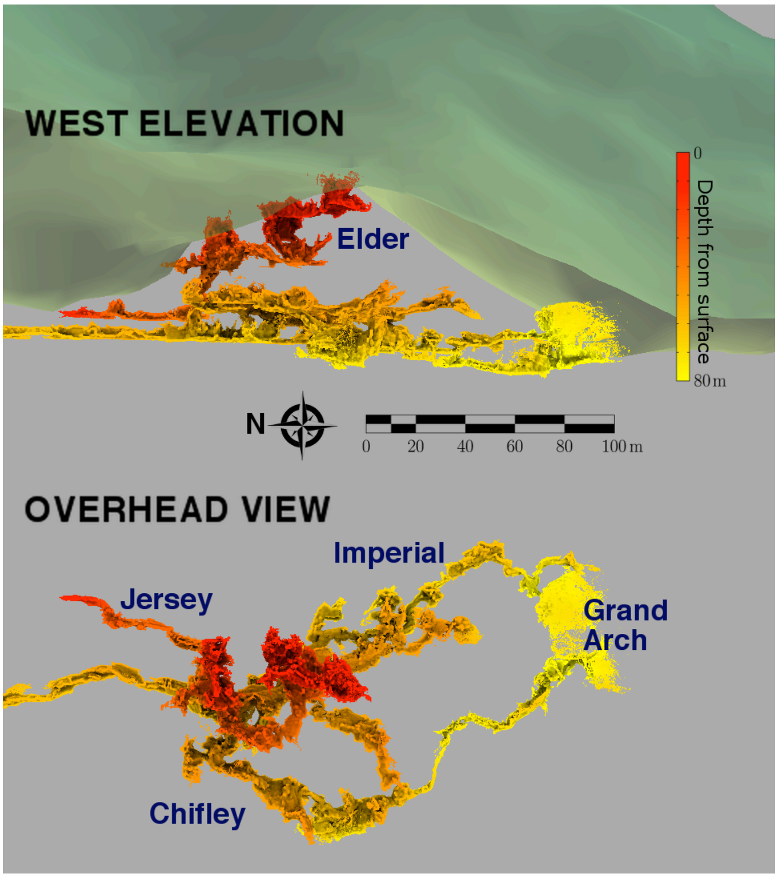

Supplementary Figure 7. Scale diagram of high-resolution 3D cave air pathway for convective ventilation. Excerpt of .mov video showing fly-through of data in 3D.

Date-time	Soil daily average Soil Rn Bq m-3	Cave daily average Cave Rn Bq m-3	Daily Soil Rn / Cave Rn	Weekly (7 days following)
13/5/16	2,132			
14/5/16	2,583			
15/5/16	2,487			
16/5/16	2,174			
17/5/16	2,251			
18/5/16	2,532	383	7	
19/5/16	2,975	314	9	13
20/5/16	2,826	583	5	
21/5/16	3,145	270	12	
22/5/16	3,063	459	7	
23/5/16	2,701	838	3	
24/5/16	3,158	84	38	
25/5/16	3,523	205	17	
26/5/16	3,654	121	30	87
27/5/16	3,919	94	42	
28/5/16	4,023	57	70	
29/5/16	4,091	77	53	
30/5/16	4,412	43	103	
31/5/16	4,113	32	127	
1/6/16	3,077	17	180	
2/6/16	3,446	27	130	128
3/6/16	3,202	19	172	
4/6/16	3,225	29	111	
5/6/16	2,665	46	58	
6/6/16	1,526	18	86	
7/6/16	2,135	11	196	
8/6/16	2,280	16	144	
9/6/16	2,324	28	83	133
10/6/16	1,878	14	136	
11/6/16	2,617	12	226	
12/6/16	3,517	22	157	
13/6/16	3,885	31	127	
14/6/16	3,632	37	97	
15/6/16	3,627	35	105	
16/6/16	3,570	36	98	

Supplementary data Table 1. Soil & Cave Rn daily & weekly average and soil / cave source ratio during the transition from summer to winter ventilation pattern. Summer ventilation draws soil gas into Chifley Cave lowering the soil Rn / cave Rn ratio. The cave chamber was flushed with ambient air to determine initial Rn & CH_4 decay rates (see Supplementary Figure 6b). The winter pattern of convective ventilation in the opposite direction draws ambient air into Chifley Cave with a low initial Rn concentration. A winter convective ventilation pattern of Chifley Cave shows a soil Rn / cave Rn ratio > 100.

	pmoA amplicon (primers A189/A682)				pmoA/amoA amplicon (primers A189/mb661)		
#OTU_ID	Pool behind door	Flitch of Bacon	Lower Katies Bower	Deep lower Katies Bower	Pool behind door	Lower Katies Bower	taxonomy
OTU1	26.041	12.593	0.054	0	0	0	k_Bacteria;p_Proteobacteria;c_Betaproteobacteria;o_Nitrosomonadales;f_Nitrosomonadaceae;g_Nitrosospira
OTU2	10.56	71.564	0.271	0	0.005	0	k_Bacteria;p_Proteobacteria;c_Alphaproteobacteria;o_Rhizobiales;f_Methylocystaceae;g_unclassifiedMethylocystaceae
OTU3	10.265	0.489	0.108	0.159	0	0	k_Bacteria;p_Proteobacteria;c_Alphaproteobacteria;o_Rhizobiales;f_Methylocystaceae;g_unclassifiedMethylocystaceae
OTU4	8.74	0	2.304	3.28	44.117	1.855	k_Bacteria;p_Proteobacteria;c_Gammaproteobacteria;o_Methylococcales;f_;g_
OTU5	0.051	0	1.192	0.688	7.003	0.22	k_Bacteria;p_Proteobacteria;c_Gammaproteobacteria;o_;f_;g_
OTU6	0.538	0	2.331	0.741	0.86	1.562	k_Bacteria;p_Proteobacteria;c_Gammaproteobacteria;o_Methylococcales;f_;g_
OTU7	0.359	0	0.136	0	14.29	2.587	k_Bacteria;p_Proteobacteria;c_Gammaproteobacteria;o_Methylococcales;f_;g_
OTU8	0.026	0	0.081	33.986	0.433	0.561	k_Bacteria;p_Proteobacteria;c_Gammaproteobacteria;o_;f_;g_
OTU9	0	0	30.136	0	0	0	k_Bacteria;p_;c_;o_;f_;g_
OTU10	0.961	0	2.629	0.238	4.222	9.151	k_Bacteria;p_Proteobacteria;c_Gammaproteobacteria;o_Methylococcales;f_;g_
OTU11	4.947	0	12.168	0	1.293	18.277	k_Bacteria;p_Proteobacteria;c_Gammaproteobacteria;o_Methylococcales;f_;g_
OTU12	0.013	0	0	0	13.33	3.148	k_Bacteria;p_Proteobacteria;c_Gammaproteobacteria;o_;f_;g_
OTU13	6.113	1.262	0.894	0	0	0	k_Bacteria;p_Proteobacteria;c_Alphaproteobacteria;o_Rhizobiales;f_Methylocystaceae;g_unclassifiedMethylocystaceae
OTU14	0.256	0.11	0.65	5.66	0	0	k_Bacteria;p_Proteobacteria;c_Alphaproteobacteria;o_Rhizobiales;f_Methylocystaceae;g_unclassifiedMethylocystaceae
OTU15	0.013	0	0	3.174	2.738	0.439	k_Bacteria;p_Proteobacteria;c_Gammaproteobacteria;o_;f_;g_
OTU16	0	0	8.374	3.332	0.071	23.524	k_Bacteria;p_Proteobacteria;c_Gammaproteobacteria;o_;f_;g_
OTU17	0	0	0	0	0.124	0.049	k_Bacteria;p_Proteobacteria;c_Alphaproteobacteria;o_;f_;g_
OTU18	0.013	0	8.347	1.296	0.147	22.474	k_Bacteria;p_Proteobacteria;c_Alphaproteobacteria;o_Rhizobiales;f_Methylocystaceae;g_unclassifiedMethylocystaceae
OTU19	13.456	0.631	0.136	4.417	0.071	0	k_Bacteria;p_Proteobacteria;c_Gammaproteobacteria;o_Methylococcales;f_;g_
OTU20	0.115	0	8.266	9.336	0.014	0.049	k_Bacteria;p_Proteobacteria;c_Gammaproteobacteria;o_;f_;g_
OTU21	0	0	0.054	0	0.124	0	k_Bacteria;p_Proteobacteria;c_Alphaproteobacteria;o_Rhizobiales;f_Beijerinckiaceae;g_
OTU22	0.243	0	0.352	0.344	0.485	0.561	k_Bacteria;p_Proteobacteria;c_Gammaproteobacteria;o_;f_;g_
OTU23	0.077	0	0.108	4.919	2.23	0.708	k_Bacteria;p_Proteobacteria;c_Gammaproteobacteria;o_Methylococcales;f_;g_
OTU24	0	0	0.081	0	6.347	3.685	k_Bacteria;p_Proteobacteria;c_Alphaproteobacteria;o_Rhizobiales;f_Methylocystaceae;g_unclassifiedMethylocystaceae
OTU25	2.115	6.722	0.163	0	0	0	k_Bacteria;p_Proteobacteria;c_Gammaproteobacteria;o_;f_;g_
OTU26	2.204	0.331	0	1.296	0	0	k_Bacteria;p_Proteobacteria;c_Gammaproteobacteria;o_Methylococcales;f_;g_
OTU27	0	0	2.358	0	0	6.955	k_Bacteria;p_Proteobacteria;c_Betaproteobacteria;o_Nitrosomonadales;f_Nitrosomonadaceae;g_Nitrosomonas
OTU28	2.499	3.188	0	0	0	0	k_Bacteria;p_;c_;o_;f_;g_
OTU29	0	0	9.973	12.14	0	0	k_Bacteria;p_Proteobacteria;c_Alphaproteobacteria;o_Rhizobiales;f_Methylocystaceae;g_unclassifiedMethylocystaceae
OTU30	3.473	0.316	0	1.111	0	0	k_Bacteria;p_Proteobacteria;c_Alphaproteobacteria;o_Rhizobiales;f_Methylocystaceae;g_unclassifiedMethylocystaceae
OTU31	3.665	0.126	0.515	1.005	0	0	k_Bacteria;p_Proteobacteria;c_Alphaproteobacteria;o_Rhizobiales;f_Methylocystaceae;g_unclassifiedMethylocystaceae
OTU32	0	0	0.407	9.601	0	0	k_Bacteria;p_Proteobacteria;c_Gammaproteobacteria;o_;f_;g_
OTU33	0	0	0	0	0.879	0.268	k_Bacteria;p_Proteobacteria;c_Alphaproteobacteria;o_Rhizobiales;f_Methylocystaceae;g_unclassifiedMethylocystaceae
OTU34	0	0	0	0	0.019	0	k_Bacteria;p_;c_;o_;f_;g_
OTU35	0.282	1.641	0.027	0	0	0	k_Bacteria;p_Proteobacteria;c_Gammaproteobacteria;o_;f_;g_
OTU36	0	0	0.054	0	0.005	0.073	k_Bacteria;p_Proteobacteria;c_Gammaproteobacteria;o_;f_;g_
OTU37	0.961	0	0.108	0.026	0.651	0.366	k_Bacteria;p_Proteobacteria;c_Gammaproteobacteria;o_;f_;g_
OTU38	0	0	0	0	0.014	0	k_Bacteria;p_Proteobacteria;c_Gammaproteobacteria;o_Methylococcales;f_;g_
OTU40	0.013	0	0	0.555	0	0	k_Bacteria;p_Proteobacteria;c_Alphaproteobacteria;o_Rhizobiales;f_Beijerinckiaceae;g_
OTU41	0.013	0	0.027	0	0.01	0.098	k_Bacteria;p_Proteobacteria;c_Gammaproteobacteria;o_Methylococcales;f_;g_
OTU42	0	0	5.583	0.185	0	0	k_Bacteria;p_Proteobacteria;c_Gammaproteobacteria;o_;f_;g_
OTU43	0	0	0.136	0.079	0.029	0.61	k_Bacteria;p_Proteobacteria;c_Gammaproteobacteria;o_Methylococcales;f_;g_
OTU44	0.013	0	0.027	0.291	0.048	0.708	k_Bacteria;p_Proteobacteria;c_Alphaproteobacteria;o_;f_;g_
OTU45	0	0	0.027	0.026	0	0.049	k_Bacteria;p_Proteobacteria;c_Gammaproteobacteria;o_;f_;g_
OTU46	0	0	0.136	0.053	0	0.488	k_Bacteria;p_Proteobacteria;c_Gammaproteobacteria;o_;f_;g_
OTU47	0	0	0	0.106	0.005	0.024	k_Bacteria;p_Proteobacteria;c_Gammaproteobacteria;o_;f_;g_
OTU48	0.026	0	0	0	0.014	0	k_Bacteria;p_Proteobacteria;c_Gammaproteobacteria;o_Methylococcales;f_;g_
OTU49	0.5	0.426	0	0	0	0	k_Bacteria;p_Proteobacteria;c_Gammaproteobacteria;o_;f_;g_
OTU50	0	0	0	0	0	0.146	k_Bacteria;p_Proteobacteria;c_Betaproteobacteria;o_Nitrosomonadales;f_Nitrosomonadaceae;g_Nitrosospira
OTU51	0	0	0.136	0	0	0	k_Bacteria;p_Proteobacteria;c_Gammaproteobacteria;o_Methylococcales;f_;g_
OTU52	0.269	0	0.867	1.428	0.01	1	k_Bacteria;p_Proteobacteria;c_Gammaproteobacteria;o_Methylococcales;f_;g_
OTU53	0.154	0.316	0	0	0	0	k_Bacteria;p_Proteobacteria;c_Gammaproteobacteria;o_Methylococcales;f_Methylococcaceae;g_
OTU54	0	0	0	0	0.024	0	k_Bacteria;p_Proteobacteria;c_Alphaproteobacteria;o_Rhizobiales;f_Methylocystaceae;g_unclassifiedMethylocystaceae
OTU55	0	0	0	0	0.09	0	k_Bacteria;p_Proteobacteria;c_Gammaproteobacteria;o_Methylococcales;f_;g_
OTU56	0.32	0	0	0	0	0	k_Bacteria;p_Proteobacteria;c_Gammaproteobacteria;o_Methylococcales;f_Crenotrichaceae;g_
OTU57	0.026	0	0	0	0.01	0	k_Bacteria;p_Proteobacteria;c_Gammaproteobacteria;o_;f_;g_
OTU58	0	0	0.081	0.079	0	0.098	k_Bacteria;p_Proteobacteria;c_Gammaproteobacteria;o_Methylococcales;f_;g_
OTU59	0.013	0.032	0	0	0	0	k_Bacteria;p_Proteobacteria;c_Gammaproteobacteria;o_;f_;g_
OTU60	0.128	0.016	0	0	0	0	k_Bacteria;p_Proteobacteria;c_Gammaproteobacteria;o_Methylococcales;f_;g_
OTU62	0	0.032	0	0.053	0	0	k_Bacteria;p_Proteobacteria;c_Gammaproteobacteria;o_;f_;g_
OTU63	0.026	0	0.081	0	0.014	0.146	k_Bacteria;p_Proteobacteria;c_Alphaproteobacteria;o_Rhizobiales;f_Methylocystaceae;g_unclassifiedMethylocystaceae
OTU64	0.038	0	0.108	0	0.01	0	k_Bacteria;p_Proteobacteria;c_Gammaproteobacteria;o_;f_;g_
OTU66	0.179	0	0	0	0	0	k_Bacteria;p_Proteobacteria;c_Gammaproteobacteria;o_Methylococcales;f_;g_
OTU67	0.013	0	0.027	0	0	0	k_Bacteria;p_;c_;o_;f_;g_
OTU69	0	0	0	0.026	0.024	0.024	k_Bacteria;p_Proteobacteria;c_Gammaproteobacteria;o_;f_;g_
OTU70	0	0	0	0	0.019	0	k_Bacteria;p_Proteobacteria;c_Alphaproteobacteria;o_Rhizobiales;f_Methylocystaceae;g_unclassifiedMethylocystaceae
OTU71	0	0	0.352	0	0	0	k_Bacteria;p_Proteobacteria;c_Gammaproteobacteria;o_Methylococcales;f_;g_
OTU72	0.038	0	0	0	0	0	k_Bacteria;p_Proteobacteria;c_;o_;f_;g_
OTU73	0	0	0	0.026	0.005	0	k_Bacteria;p_Proteobacteria;c_Alphaproteobacteria;o_Rhizobiales;f_Methylocystaceae;g_unclassifiedMethylocystaceae
OTU74	0	0	0	0	0.014	0.049	k_Bacteria;p_Proteobacteria;c_Gammaproteobacteria;o_;f_;g_
OTU75	0	0	0	0.185	0	0	k_Bacteria;p_Proteobacteria;c_Alphaproteobacteria;o_Rhizobiales;f_Methylocystaceae;g_unclassifiedMethylocystaceae
OTU77	0.167	0	0	0	0	0	k_Bacteria;p_Proteobacteria;c_Gammaproteobacteria;o_;f_;g_
OTU78	0	0	0.136	0	0	0	k_Bacteria;p_Proteobacteria;c_Gammaproteobacteria;o_;f_;g_
OTU79	0.026	0.032	0	0	0	0	k_Bacteria;p_Proteobacteria;c_Gammaproteobacteria;o_;f_;g_
OTU80	0.038	0	0	0	0.029	0	k_Bacteria;p_Proteobacteria;c_Gammaproteobacteria;o_;f_;g_
OTU81	0.013	0.016	0	0	0	0	k_Bacteria;p_Proteobacteria;c_Alphaproteobacteria;o_Rhizobiales;f_Methylocystaceae;g_unclassifiedMethylocystaceae
OTU82	0	0	0	0	0.076	0	k_Bacteria;p_Proteobacteria;c_Gammaproteobacteria;o_;f_;g_
OTU84	0	0.032	0	0.026	0	0	k_Bacteria;p_Proteobacteria;c_Alphaproteobacteria;o_Rhizobiales;f_Methylocystaceae;g_unclassifiedMethylocystaceae
OTU85	0	0	0	0.026	0.014	0	k_Bacteria;p_Proteobacteria;c_Gammaproteobacteria;o_Methylococcales;f_;g_
OTU88	0	0	0	0	0	0.049	k_Bacteria;p_Proteobacteria;c_;o_;f_;g_
OTU89	0	0	0	0	0.014	0	k_Bacteria;p_Proteobacteria;c_Gammaproteobacteria;o_Methylococcales;f_;g_
OTU91	0	0	0	0	0.024	0	k_Bacteria;p_Proteobacteria;c_Gammaproteobacteria;o_;f_;g_
OTU92	0	0	0	0.026	0.019	0	k_Bacteria;p_Proteobacteria;c_Gammaproteobacteria;o_;f_;g_
OTU93	0	0.032	0	0	0	0	k_Bacteria;p_Proteobacteria;c_Gammaproteobacteria;o_;f_;g_
OTU94	0	0	0	0	0.014	0	k_Bacteria;p_Proteobacteria;c_Alphaproteobacteria;o_Rhizobiales;f_;g_
OTU96	0	0.095	0	0	0	0	k_Bacteria;p_;c_;o_;f_;g_
OTU101	0	0	0	0.026	0.014	0	k_Bacteria;p_Proteobacteria;c_Gammaproteobacteria;o_;f_;g_
OTU103	0	0	0	0.053	0	0	k_Bacteria;p_Proteobacteria;c_Gammaproteobacteria;o_;f_;g_

Supplementary data Table 2 Proportions of different *pmoA* sequences in different parts of Chifley Cave. Amplicon sequences (4,000-20,000 sequences per sample) were obtained by 454-sequencing after amplification of cave DNA with primers A189/A682 [31] or [36]. Values given are percentages of each OTU within each sample.

Methane depletion rates (laboratory)	
**Sample location**	**Methane depletion rate**
	nmol g-1 soil h-1
Upper Katie's Bower	1.06 ± 0.2
Lower Katie's Bower	1.21 ± 0.27
Flitch of Bacon	3.06 ± 0.46
Pool behind door	3.93 ± 0.23
Niche outside internal door	0.81 ± 0.07

Supplementary data Table 3 Methane depletion rates in cave soils; rate of methane consumption from an initial 1,000 ppm incubated in closed vial (120 mL) with 5 g soil.

	Katies Bower to Elder	% of total cave path	
Start (x,y,z) 38.7 43.13 63.57 m			
End (x,y,z) 5.435 -2.205 -11.11 m			
Raw path length m	363.7	67.9	
Smoothed path length m	268.3	69.8	
Total surface area (res 0.05m) m2	19,714.2	81.4	
Volume (res 0.05m) m3	6,891.2	71.9	
Katies Bower to Grand Arch		% of total cave path	Total Path / area / volume
Raw path length m	172.1	32.1	535.8
Smoothed path length m	116.1	30.2	384.5
Total surface area (res 0.03m) m2	4,496.4	18.6	24,210.6
Volume (res 0.03m) m3	2,696.2	28.1	9,587.4
Chifley entrance to Grand Arch elevation m AHD	765.4		
Chifley entrance to Elder opening elevation m AHD	827.4		
Chifley entrance to Elder opening elevation difference	62.0		

Supplementary data Table 4 Path length and area measured to either entrance.
